# Supplementary material for: The effect of air-pollution and weather exposure on mortality and hospital admission and implications for further research: A systematic scoping review
Source: PLoS One. 2020 Oct 29;15(10):e0241415. doi: 10.1371/journal.pone.0241415 (PMC7595412; doi:10.1371/journal.pone.0241415)
Supplement: S1 Table — (DOCX) [file pone.0241415.s002.docx]

**Title:** The Effect of Air-pollution and weather exposures on Mortality and Hospital Admission and implications for further research: A Systematic Scoping Review

**S1 Table**

The search codes used in PubMed and Web of Science databases for this scoping literature review

| PubMed | (("air-pollution"[MeSH Terms] OR "weather"[MeSH Terms] OR "climate"[MeSH Terms] OR "climate change"[MeSH Terms] OR "extreme cold weather"[MeSH Terms] OR "cold climate"[MeSH Terms] OR "extreme hot weather"[MeSH Terms] OR "extreme-weather"[MeSH Terms] OR "Air Quality"[All Fields]) AND ("mortality"[MeSH Terms] OR "hospital mortality"[MeSH Terms] OR (("hospitals"[MeSH Terms] OR "hospitals"[All Fields] OR "hospital"[All Fields]) AND (Admission[All Fields] OR Entry[All Fields])) AND (("europe"[MeSH Terms] OR "europe"[All Fields]) OR Britain[All Fields] OR UK[All Fields] OR "United Kingdom"[All Fields] OR ("scotland"[MeSH Terms] OR "scotland"[All Fields]) OR ("england"[MeSH Terms] OR "england"[All Fields]) OR ("wales"[MeSH Terms] OR "wales"[All Fields])))) NOT ("influenza, human"[MeSH Terms] OR ("influenza"[All Fields] AND "human"[All Fields]) OR "human influenza"[All Fields] OR "influenza"[All Fields]) NOT (“indoor”) NOT (“forecasting”) NOT (“China”) NOT (“health impact assessment”) NOT (“ecological”) NOT (“cross-sectional”) |
| --- | --- |
| Web of Science | ((air AND pollution) OR weather OR climate OR (Air AND Quality)) AND (mortality OR ((hospitals OR hospital) AND (Admission OR Entry))) AND (Europe OR Britain OR UK OR (United AND Kingdom) OR Scotland OR England OR Wales) NOT ((influenza AND human) OR influenza) NOT (indoor) NOT (forecasting) NOT (China) NOT (health AND impact AND assessment) NOT (ecological) NOT (cross-sectional) |
