# Supplementary material for: The effect of air-pollution and weather exposure on mortality and hospital admission and implications for further research: A systematic scoping review
Source: PLoS One. 2020 Oct 29;15(10):e0241415. doi: 10.1371/journal.pone.0241415 (PMC7595412; doi:10.1371/journal.pone.0241415)
Supplement: S2 Table — (DOCX) [file pone.0241415.s003.docx]

**Title:** The Effect of Air-pollution and weather exposures on Mortality and Hospital Admission and implications for further research: A Systematic Scoping Review

**S2 Table**

S2 Table includes the list of the 112 reviewed studies and the Tables that summarise the characteristics of the included studies in more details by the type of investigated health outcome.

**Studies involved in the final review that include both mortality and hospital admission health outcomes (n=12)**

| Study reference number | Study Period | Location | Study design | Exposures | Health Outcome(s) | |
| --- | --- | --- | --- | --- | --- | --- |
|  |  |  |  |  | **Mortality** | **Hospital admission** |
| (1) | 2003-2006 | Italy | Time-series | NO2, PM10 | All-cause, cardiovascular, respiratory | Cardiovascular, cerebrovascular, respiratory |
| (2) | 2002-2006 | Czech Republic | Time-series | O3 | All-cause, cardiovascular, respiratory | Cardiovascular, respiratory |
| (3) | 2001-2010 | Europe | Case-crossover | PM10 | All-cause, cardiovascular, respiratory | Cardiovascular, respiratory |
| (4) | 2002-2007 | Portugal | Time-series | Temperature | All-cause | All-cause, cardiovascular, respiratory |
| (5) | 1990-2002 | Sweden | Case-crossover | Temperature | All-cause | All-cause, psychiatric |
| (6) | 1993-2006 | England | Time-series | Temperature | All-cause | All-cause, cardiovascular, respiratory |
| (7) | 2003-2010 | England | Cohort | PM10, PM2.5, NOx, NO2, O3 | All-cause | All-cause |
| (8) | 2003-2013 | Spain and Italy | Case-crossover | PM10, PM2.5 | All-cause, cardiovascular, respiratory | Cardiovascular, respiratory |
| (9) | 2001-2013 | Spain | Case-crossover | Temperature, humidity, NO2, SO2, O3, PM10, CO | Pulmonary embolism | Pulmonary embolism |
| (10) | 2003-2008 | England and Wales | Case-crossover | PM10, PM2.5, CO, NO2, SO2, O3 | Cardiovascular, myocardial infarction, stroke, ischemic heart disease, chronic ischemic heart, arrhythmias, atrial fibrillation, atrioventricular conduction, pulmonary embolism, heart failure | Cardiovascular, myocardial infarction, stroke, ischemic heart disease, chronic ischemic heart, arrhythmias, atrial fibrillation, atrioventricular conduction, pulmonary embolism, heart failure |
| (11) | 2001-2013 | Spain | Time-series | Temperature, O3, NO2, SO2, PM10 | Ischemic stroke | Ischemic stroke |
| (12) | 1999-2000 | France | Time-series | PM10, PM2.5, NO2, O3 | All-cause, cardiovascular, respiratory | All-cause, cardiovascular, respiratory |

**Studies involved in the final review that include mortality health outcomes (n=70)**

| Study reference number | Study period | Location | Study design | Exposures | Health outcome(s) |
| --- | --- | --- | --- | --- | --- |
| (13) | 2001-2010 | Italy | Cohort | PM10, PM2.5 | All-cause, cardiovascular, ischemic heart disease |
| (14) | 2008-2011 | Europe | Cohort | PM10, PM2.5, NO2, NOx | All-cause |
| (15) | 2008-2011 | Europe | Cohort | PM10, PM2.5, NO2, NOx | Cardiovascular, ischemic heart disease myocardial infarction, cerebrovascular |
| (16) | 2003-2007 | England | Cohort | PM10, PM2.5, NO2, O3, SO2 | All-cause, cardiovascular, respiratory, lung cancer |
| (17) | 2001-2010 | Italy | Cohort | PM2.5, NO2 | All-cause, Cardiovascular, ischemic heart disease, cerebrovascular, respiratory, lung cancer |
| (18) | 1989-2015 | England | Cohort | SO2, NO2, NOx, PM10, PM2.5, black smoke | Cardiovascular |
| (19) | 2008-2011 | Europe | Cohort | PM10, PM2.5, NO2, NOx | Respiratory |
| (20) | 2004-2011 | Netherland | Cohort | PM10, NO2 | All-cause, respiratory, lung cancer, cardiovascular |
| (21) | 1971-2009 | England and Wales | Cohort | PM10, SO2, black smoke | All-cause, cardiovascular, respiratory |
| (22) | 1993-1997 | Denmark | Cohort | PM2.5, PM10, NO2, O3, black carbon | All-cause, respiratory, cardiovascular |
| (23) | 2008-2009 | Spain | Cohort | PM10, PM2.5, NO2 | All-cause |
| (24) | 1993/1997-2009 | Denmark | Cohort | NO2 | All-cause, cardiovascular, ischemic heart disease, cardiac rhythm disturbances, heart failure, cerebrovascular |
| (25) | 1973-2007 | Sweden | Cohort | NOx | All-cause, cardiovascular, ischemic heart disease, acute myocardial infarction, cerebrovascular, respiratory |
| (26) | 2004-2010 | England and Wales | Cohort | PM10, PM2.5, NO2, NOx | All-cause |
| (27) | 1980-2015 | Spain | Time-series | Temperature | Cardiovascular, respiratory |
| (28) | 2006-2010 | Italy | Case-crossover | PM10, PM2.5 | All-cause |
| (29) | 2004-2010 | Europe | Time-series | Temperature, PM10, NO2, O3 | All-cause, cardiovascular, respiratory |
| (30) | 1990-2004 | Europe | Time-series | Temperature, O3, PM10 | All-cause, cardiovascular, respiratory |
| (31) | 1997-2013 | Estonia | Time-series | Temperature | All-cause |
| (32) | 1901-2009 | Sweden | Time-series | Temperature | All-cause |
| (33) | 1997-2015 in Estonia & 2009-2015 in Latvia | Estonia and Latvia | Time-series | Temperature | All-cause, cardiovascular, respiratory, external cause |
| (34) | 2011-2012 | England | Time-series | NOx, CO, elemental carbon, black carbon, copper, zinc, aluminium | All-cause, cardiovascular, respiratory |
| (35) | 2004-2009 | France | Time-series | Temperature, NO2 | All-cause |
| (36) | 1990-2006 | Germany | Time-series | Temperature | All-cause, cardiovascular, respiratory |
| (37) | 1998-2010 | Germany | Time-series | PM10, O3, temperature | All-cause |
| (38) | 1990-2006 | Germany | Time-series | Temperature | Cardiovascular, ischemic heart disease, cerebrovascular |
| (39) | 2004-2009 | France | Case-crossover | NO2 | All-cause |
| (40) | 2001-2010 | Italy | Case-crossover | PM10 | All-cause, cardiovascular, COPD |
| (41) | 2004-2005 | Spain | Case-crossover | Temperature | Cardiovascular, acute myocardial infarction, respiratory, cancer |
| (42) | 2003-2006 | Spain | Time-series | Temperature | All-cause |
| (43) | 2011-2012 (Germany), 2012-2013 (Slovenia & Czech Republic), 2013-2014 (Ukraine) | Germany, Slovenia, Czech Republic, and Ukraine | Time-series | PM10, PM2.5, NO2 | All-cause, cardiovascular, respiratory |
| (44) | 2000-2009 | Spain | Time-series | NO2 | All-cause, cardiovascular, respiratory |
| (45) | 2000-2009 | Spain | Time-series | Temperature | All-cause, cardiovascular, respiratory |
| (46) | 1992-2001 | Norway | Case-crossover | PM2.5, NO2 | All-cause, cardiovascular, respiratory |
| (47) | 1992-2015 | Spain | Time-series | Temperature | All-cause |
| (48) | 2000-2008 | Sweden | Time-series | PM2.5, PM10, CO, O3 | All-cause |
| (49) | 1975-2008 | Spain | Time-series | Temperature | All-cause, cardiovascular, respiratory |
| (50) | 1990-2005 | Austria | Time-series | PM10, PM2.5, NO2 | All-cause |
| (51) | 2000-2016 | Sweden | Time-series | PM10, PM2.5, O3, NO2, black carbon | All-cause |
| (52) | 1997-2013 | Estonia | Time-series | Temperature | External cause |
| (53) | 1997-2013 | Estonia | Time-series | Temperature | All-cause |
| (54) | 2000-2008 | Italy | Time-series | Temperature | All-cause, congestive heart failure, COPD, diabetes, psychiatric, myocardial infarction |
| (55) | 2000-2006 | France | Time-series | PM10, PM2.5 | All-cause, cardiovascular, cardiac, ischemic heart disease, cerebrovascular, respiratory |
| (56) | 2000-2010 | France | Time-series | Temperature | All-cause |
| (57) | 2003-2007 | Spain | Case-crossover | PM1, PM10, PM2.5 | Cardiovascular, respiratory, cerebrovascular |
| (58) | 2008-2013 | Poland | Time-series | Temperature | All-cause |
| (59) | 1995-2013 | Switzerland | Time-series | Temperature | All-cause |
| (60) | 2006-2012 | Italy | Time-series | PM10 | All-cause, cardiovascular, respiratory |
| (61) | 1998-2014 | Italy | Time-series | PM10, PM2.5, NO2, CO, SO2 | All-cause |
| (62) | 2001-2010 | Southern Europe | Time-series | PM10, PM2.5 | Diabetes, cardiovascular, cerebrovascular, lower respiratory tract infection, COPD |
| (63) | 1983-2013 | Spain | Time-series | Evapotranspiration & Precipitation indexes | All-cause, cardiovascular, respiratory |
| (64) | 1999-2005 | England | Time-series | Temperature | All-cause |
| (65) | 2001-2010 | Mediterranean Europe | Time-series | PM10, PM2.5 | All-cause, cardiovascular, respiratory |
| (66) | 1749-1859 | Sweden | Time-series | Temperature, rainfall | All-cause |
| (67) | 1990-2010 | Europe | Time-series | Temperature | All-cause |
| (68) | 2009-2014 | Serbia | Time-series | Temperature | Cardiovascular, respiratory |
| (69) | 1990-2004 | Spain | Time-series | Temperature | All-cause |
| (70) | 1999-2013 | Europe | Time-series | Particulate matter (PM) | All-cause, cardiovascular, respiratory |
| (71) | 2006-2010 | Italy | Time-series | Temperature | All-cause |
| (72) | 2009-2014 | Serbia | Time-series | PM10, NO2, SO2, soot | All-cause, cardiovascular, respiratory |
| (73) | 1995-2009 | Netherlands | Case-crossover | Temperature, PM10, NO2, O3 | All-cause |
| (74) | 2000-2005 | England | Time-series | Ox (O3 + NO2), O3, NO2 | All-cause |
| (75) | 2000-2012 | Greece | Time-series | Temperature | All-cause, cardiovascular, respiratory |
| (76) | 1999-2006 | Spain | Case-crossover | Temperature | All-cause |
| (77) | 1984-2007 | Republic of Ireland & Northern Ireland | Case-crossover | Temperature | All-cause, cardiovascular, respiratory, stroke |
| (78) | 1993-2006 | England and Wales | Time-series | Temperature | All-cause |
| (79) | 1993-2006 | England and Wales | Time-series | Temperature | All-cause |
| (80) | 2002-2011 | Ireland | Time-series | Temperature | All-cause |
| (81) | 2002-2011 | Ireland | Cohort | Temperature, PM10, SO2, NOx | All-cause |
| (82) | 1996-2006 | England | Case-crossover | PM10, SO2, CO, NO2, O3 | Sudden infant death |

**Studies involved in the final review that include hospital admission health outcomes (n=30)**

| Study reference number | Study period | Location | Study design | Exposures | Health outcome(s) |
| --- | --- | --- | --- | --- | --- |
| (83) | 2001-2010 | Scotland | Time-series | Temperature | COPD |
| (84) | 1995-2012 | Denmark | Cohort | Sunshine hours, ultraviolet dose, temperature, rainfall, snow-cover | Psychiatric (mania) |
| (85) | 2004-2011 | France | Time-series | CO, temperature, wind speed | Vaso-occlusive painful crises resulting in chest disease |
| (86) | 1987-1998 | Italy | Time-series | Temperature, humidity | Angina pectoris |
| (87) | 2005-2009 | Portugal | Time-series | PM10, PM2.5, O3 | Respiratory |
| (88) | 2001-2009 | Italy | Time-series | PM10 | Multiple sclerosis |
| (89) | 2003-2009 | England & Wales | Case-crossover | Temperature | Myocardial infarction |
| (90) | 2010-2011 | Serbia | Time-series | Temperature, humidity, air pressure | Angina pectoris, essential hypertension, acute myocardial infarction, ischemic heart disease |
| (91) | 2008-2010 | England | Case-crossover | PM10 | COPD, asthma |
| (92) | 2001-2005 | Italy | Case-crossover | PM10 | Cardiovascular, acute coronary syndrome, arrhythmias, conduction disorder, heart failure |
| (93) | 2008-2011 | Belgium | Time-series | NO2 | Cardiovascular, arrhythmia, acute myocardial infarction, ischemic heart disease, haemorrhagic stroke |
| (94) | 1991-2003 | Netherlands | Cohort | PM10, NO2, elemental carbon | Ischemic heart disease, cerebrovascular |
| (95) | 2011–2012 (Germany),  2012–2013 (Slovenia & Czech Republic), 2013–2014 (Ukraine) | Germany, Slovenia, Czech Republic, Ukraine | Time-series | PM10, PM2.5, NO2 | Cardiovascular, respiratory, diabetes |
| (96) | 2001-2011 | Germany | Time-series | PM10, temperature | All-cause |
| (97) | 1971-2002 | Ireland | Time-series | Wind speed and direction, barometric pressure, sunshine, sunlight radiation, rainfall, temperature | Psychiatric |
| (98) | 1971-2002 | Ireland | Time-series | Wind speed and direction, barometric pressure, sunshine, sunlight radiation, rainfall, temperature | Psychiatric (mania and depression) |
| (99) | 2006-2013 | Spain | Self-controlled case series | Temperature | Cardiovascular, coronary heart disease, stroke, heart failure |
| (100) | 2001-2010 | Norway | Time-series | Daylight, sunshine, temperature, rainfall | Trauma |
| (101) | 2001-2015 | Spain | Time-series | Temperature, CO, NO2, SO2, PM10 | Acute myocardial |
| (102) | 2001-2012 | England | Time-series | NOx, CO | Cardiovascular, respiratory |
| (103) | 2003-2014 | Spain | Time-series | NO2, SO2, CO, O3, PM10, wind speed, humidity, temperature, air pressure, rainfall, solar radiation | Stroke |
| (104) | 2001-2010 | Europe | Time-series | PM10, PM2.5 | Cardiovascular, respiratory |
| (105) | 2010-2012 | Czech Republic | Time-series | PM10 | Cardiovascular, respiratory |
| (106) | 2002-2007 | The Netherlands | Time-series | Temperature | Potential heat-related diseases, respiratory, cardiovascular, fractures of femur urgent emergency room |
| (107) | 1985–2010 | Sweden | Case-crossover | Temperature | Acute myocardial infarction |
| (108) | 2006-2016 | Poland | Time-series | PM10, PM2.5 | COPD |
| (109) | 2002-2010 | Poland | Time-series | Sunshine | Psychiatric |
| (110) | 2011-2012 | Italy | Time-series | Temperature | Emergency |
| (111) | 2003-2007 | England | Time-series | NO2, O3 | Respiratory |
| (112) | 2008-2012 | England and France | Time-series | Wind speed, rainfall, barometric pressure | Sickle cell disease |

**The list of reviewed articles**

1. Carugno M, Consonni D, Randi G, Catelan D, Grisotto L, Bertazzi PA, et al. Air pollution exposure, cause-specific deaths and hospitalizations in a highly polluted Italian region. Environmental Research. 2016;147:415-24.

2. Hunova I, Maly M, Rezacova J, Branis M. Association between ambient ozone and health outcomes in Prague. International Archives of Occupational and Environmental Health. 2013;86(1):89-97.

3. Stafoggia M, Zauli-Sajani S, Pey J, Samoli E, Alessandrini E, Basagana X, et al. Desert Dust Outbreaks in Southern Europe: Contribution to Daily PM10 Concentrations and Short-Term Associations with Mortality and Hospital Admissions. Environmental Health Perspectives. 2016;124(4):413-9.

4. Monteiro A, Carvalho V, Oliveira T, Sousa C. Excess mortality and morbidity during the July 2006 heat wave in Porto, Portugal. Int J Biometeorol. 2013;57(1):155-67.

5. Rocklov J, Forsberg B, Ebi K, Bellander T. Susceptibility to mortality related to temperature and heat and cold wave duration in the population of Stockholm County, Sweden. Glob Health Action. 2014;7:22737.

6. Hajat S, Chalabi Z, Wilkinson P, Erens B, Jones L, Mays N. Public health vulnerability to wintertime weather: time-series regression and episode analyses of national mortality and morbidity databases to inform the Cold Weather Plan for England. Public Health. 2016;137:26-34.

7. Tonne C, Halonen JI, Beevers SD, Dajnak D, Gulliver J, Kelly FJ, et al. Long-term traffic air and noise pollution in relation to mortality and hospital readmission among myocardial infarction survivors. Int J Hyg Environ Health. 2016;219(1):72-8.

8. Basagana X, Jacquemin B, Karanasiou A, Ostro B, Querol X, Agis D, et al. Short-term effects of particulate matter constituents on daily hospitalizations and mortality in five South-European cities: Results from the MED-PARTICLES project. Environment International. 2015;75:151-8.

9. de Miguel-Diez J, Jimenez-Garcia R, Lopez de Andres A, Hernandez-Barrera V, Carrasco-Garrido P, Monreal M, et al. Analysis of environmental risk factors for pulmonary embolism: A case-crossover study (2001-2013). Eur J Intern Med. 2016;31:55-61.

10. Milojevic A, Wilkinson P, Armstrong B, Bhaskaran K, Smeeth L, Hajat S. Short-term effects of air pollution on a range of cardiovascular events in England and Wales: case-crossover analysis of the MINAP database, hospital admissions and mortality. Heart. 2014;100(14):1093-8.

11. Roye D, Zarrabeitia MT, Riancho J, Santurtun A. A time series analysis of the relationship between apparent temperature, air pollutants and ischemic stroke in Madrid, Spain. Environ Res. 2019;173:349-58.

12. Sanyal S, Rochereau T, Maesano CN, Com-Ruelle L, Annesi-Maesano I. Long-Term Effect of Outdoor Air Pollution on Mortality and Morbidity: A 12-Year Follow-Up Study for Metropolitan France. Int J Environ Res Public Health. 2018;15(11).

13. Badaloni C, Cesaroni G, Cerza F, Davoli M, Brunekreef B, Forastiere F. Effects of long-term exposure to particulate matter and metal components on mortality in the Rome longitudinal study. Environ Int. 2017;109:146-54.

14. Beelen R, Raaschou-Nielsen O, Stafoggia M, Andersen ZJ, Weinmayr G, Hoffmann B, et al. Effects of long-term exposure to air pollution on natural-cause mortality: an analysis of 22 European cohorts within the multicentre ESCAPE project. Lancet. 2014;383(9919):785-95.

15. Beelen R, Stafoggia M, Raaschou-Nielsen O, Andersen ZJ, Xun WW, Katsouyanni K, et al. Long-term Exposure to Air Pollution and Cardiovascular Mortality An Analysis of 22 European Cohorts. Epidemiology. 2014;25(3):368-78.

16. Carey IM, Atkinson RW, Kent AJ, van Staa T, Cook DG, Anderson HR. Mortality Associations with Long-Term Exposure to Outdoor Air Pollution in a National English Cohort. American Journal of Respiratory and Critical Care Medicine. 2013;187(11):1226-33.

17. Cesaroni G, Badaloni C, Gariazzo C, Stafoggia M, Sozzi R, Davoli M, et al. Long-term exposure to urban air pollution and mortality in a cohort of more than a million adults in Rome. Environ Health Perspect. 2013;121(3):324-31.

18. Dehbi HM, Blangiardo M, Gulliver J, Fecht D, de Hoogh K, Al-Kanaani Z, et al. Air pollution and cardiovascular mortality with over 25 years follow-up: A combined analysis of two British cohorts. Environment International. 2017;99:275-81.

19. Dimakopoulou K, Samoli E, Beelen R, Stafoggia M, Andersen ZJ, Hoffmann B, et al. Air Pollution and Nonmalignant Respiratory Mortality in 16 Cohorts within the ESCAPE Project. American Journal of Respiratory and Critical Care Medicine. 2014;189(6):684-96.

20. Fischer PH, Marra M, Ameling CB, Hoek G, Beelen R, de Hoogh K, et al. Air Pollution and Mortality in Seven Million Adults: The Dutch Environmental Longitudinal Study (DUELS). Environmental Health Perspectives. 2015;123(7):697-704.

21. Hansell A, Ghosh RE, Blangiardo M, Perkins C, Vienneau D, Goffe K, et al. Historic air pollution exposure and long-term mortality risks in England and Wales: prospective longitudinal cohort study. Thorax. 2016;71(4):330-8.

22. Hvidtfeldt UA, Sorensen M, Geels C, Ketzel M, Khan J, Tjonneland A, et al. Long-term residential exposure to PM2.5, PM10, black carbon, NO2, and ozone and mortality in a Danish cohort. Environ Int. 2019;123:265-72.

23. Nieuwenhuijsen MJ, Gascon M, Martinez D, Ponjoan A, Blanch J, Garcia-Gil MD, et al. Air Pollution, Noise, Blue Space, and Green Space and Premature Mortality in Barcelona: A Mega Cohort. International Journal of Environmental Research and Public Health. 2018;15(11).

24. Raaschou-Nielsen O, Andersen ZJ, Jensen SS, Ketzel M, Sorensen M, Hansen J, et al. Traffic air pollution and mortality from cardiovascular disease and all causes: a Danish cohort study. Environ Health. 2012;11:60.

25. Stockfelt L, Andersson EM, Molnar P, Rosengren A, Wilhelmsen L, Sallsten G, et al. Long term effects of residential NO(x) exposure on total and cause-specific mortality and incidence of myocardial infarction in a Swedish cohort. Environ Res. 2015;142:197-206.

26. Tonne C, Wilkinson P. Long-term exposure to air pollution is associated with survival following acute coronary syndrome. European Heart Journal. 2013;34(17):1306-11.

27. Achebak H, Devolder D, Ballester J. Heat-related mortality trends under recent climate warming in Spain: A 36-year observational study. PLoS Med. 2018;15(7):e1002617.

28. Alessandrini ER, Stafoggia M, Faustini A, Berti G, Canova C, De Togni A, et al. Association Between Short-Term Exposure to PM2.5 and PM10 and Mortality in Susceptible Subgroups: A Multisite Case-Crossover Analysis of Individual Effect Modifiers. Am J Epidemiol. 2016;184(10):744-54.

29. Analitis A, de' Donato F, Scortichini M, Lanki T, Basagana X, Ballester F, et al. Synergistic Effects of Ambient Temperature and Air Pollution on Health in Europe: Results from the PHASE Project. International Journal of Environmental Research and Public Health. 2018;15(9).

30. Analitis A, Michelozzi P, D'Ippoliti D, De'Donato F, Menne B, Matthies F, et al. Effects of heat waves on mortality: effect modification and confounding by air pollutants. Epidemiology. 2014;25(1):15-22.

31. Aring;strom DO, Aring;strom C, Rekker K, Indermitte E, Orru H. High Summer Temperatures and Mortality in Estonia. Plos One. 2016;11(5).

32. Astrom DO, Forsberg B, Edvinsson S, Rocklov J. Acute fatal effects of short-lasting extreme temperatures in Stockholm, Sweden: evidence across a century of change. Epidemiology. 2013;24(6):820-9.

33. Astrom DO, Veber T, Martinsone Z, Kaluznaja D, Indermitte E, Oudin A, et al. Mortality Related to Cold Temperatures in Two Capitals of the Baltics: Tallinn and Riga. Medicina-Lithuania. 2019;55(8).

34. Atkinson RW, Analitis A, Samoli E, Fuller GW, Green DC, Mudway IS, et al. Short-term exposure to traffic-related air pollution and daily mortality in London, UK. Journal of Exposure Science and Environmental Epidemiology. 2016;26(2):125-32.

35. Benmarhnia T, Oulhote Y, Petit C, Lapostolle A, Chauvin P, Zmirou-Navier D, et al. Chronic air pollution and social deprivation as modifiers of the association between high temperature and daily mortality. Environ Health. 2014;13(1):53.

36. Breitner S, Wolf K, Devlin RB, Diaz-Sanchez D, Peters A, Schneider A. Short-term effects of air temperature on mortality and effect modification by air pollution in three cities of Bavaria, Germany: a time-series analysis. Sci Total Environ. 2014;485-486:49-61.

37. Burkart K, Canario P, Breitner S, Schneider A, Scherber K, Andrade H, et al. Interactive short-term effects of equivalent temperature and air pollution on human mortality in Berlin and Lisbon. Environ Pollut. 2013;183:54-63.

38. Breitner S, Wolf K, Peters A, Schneider A. Short-term effects of air temperature on cause-specific cardiovascular mortality in Bavaria, Germany. Heart. 2014;100(16):1272-80.

39. Deguen S, Petit C, Delbarre A, Kihal W, Padilla C, Benmarhnia T, et al. Neighbourhood Characteristics and Long-Term Air Pollution Levels Modify the Association between the Short-Term Nitrogen Dioxide Concentrations and All-Cause Mortality in Paris. PLoS One. 2015;10(7):e0131463.

40. Faustini A, Stafoggia M, Renzi M, Cesaroni G, Alessandrini E, Davoli M, et al. Does chronic exposure to high levels of nitrogen dioxide exacerbate the short-term effects of airborne particles? Occup Environ Med. 2016;73(11):772-8.

41. Gomez-Acebo I, Llorca J, Dierssen T. Cold-related mortality due to cardiovascular diseases, respiratory diseases and cancer: a case-crossover study. Public Health. 2013;127(3):252-8.

42. Gomez-Acebo I, Llorca J, Rodriguez-Cundin P, Dierssen-Sotos T. Extreme temperatures and mortality in the North of Spain. Int J Public Health. 2012;57(2):305-13.

43. Lanzinger S, Schneider A, Breitner S, Stafoggia M, Erzen I, Dostal M, et al. Associations between ultrafine and fine particles and mortality in five central European cities - Results from the UFIREG study. Environ Int. 2016;88:44-52.

44. Linares C, Falcon I, Ortiz C, Diaz J. An approach estimating the short-term effect of NO2 on daily mortality in Spanish cities. Environ Int. 2018;116:18-28.

45. Lopez-Bueno JA, Diaz J, Linares C. Differences in the impact of heat waves according to urban and peri-urban factors in Madrid. Int J Biometeorol. 2019;63(3):371-80.

46. Madsen C, Rosland P, Hoff DA, Nystad W, Nafstad P, Naess OE. The short-term effect of 24-h average and peak air pollution on mortality in Oslo, Norway. Eur J Epidemiol. 2012;27(9):717-27.

47. Mari-Dell'Olmo M, Tobias A, Gomez-Gutierrez A, Rodriguez-Sanz M, Garcia de Olalla P, Camprubi E, et al. Social inequalities in the association between temperature and mortality in a South European context. Int J Public Health. 2019;64(1):27-37.

48. Meister K, Johansson C, Forsberg B. Estimated short-term effects of coarse particles on daily mortality in Stockholm, Sweden. Environ Health Perspect. 2012;120(3):431-6.

49. Miron IJ, Linares C, Montero JC, Criado-Alvarez JJ, Diaz J. Changes in cause-specific mortality during heat waves in central Spain, 1975-2008. Int J Biometeorol. 2015;59(9):1213-22.

50. Neuberger M, Moshammer H, Rabczenko D. Acute and subacute effects of urban air pollution on cardiopulmonary emergencies and mortality: time series studies in Austrian cities. Int J Environ Res Public Health. 2013;10(10):4728-51.

51. Olstrup H, Johansson C, Forsberg B, Astrom C. Association between Mortality and Short-Term Exposure to Particles, Ozone and Nitrogen Dioxide in Stockholm, Sweden. Int J Environ Res Public Health. 2019;16(6).

52. Orru H, Astrom DO. Increases in external cause mortality due to high and low temperatures: evidence from northeastern Europe. Int J Biometeorol. 2017;61(5):963-6.

53. Oudin Astrom D, Astrom C, Rekker K, Indermitte E, Orru H. High Summer Temperatures and Mortality in Estonia. PLoS One. 2016;11(5):e0155045.

54. Oudin Astrom D, Schifano P, Asta F, Lallo A, Michelozzi P, Rocklov J, et al. The effect of heat waves on mortality in susceptible groups: a cohort study of a mediterranean and a northern European City. Environ Health. 2015;14:30.

55. Pascal M, Falq G, Wagner V, Chatignoux E, Corso M, Blanchard M, et al. Short-term impacts of particulate matter (PM10, PM10-2.5, PM2.5) on mortality in nine French cities. Atmospheric Environment. 2014;95:175-84.

56. Pascal M, Wagner V, Corso M, Laaidi K, Ung A, Beaudeau P. Heat and cold related-mortality in 18 French cities. Environ Int. 2018;121(Pt 1):189-98.

57. Perez L, Tobias A, Querol X, Pey J, Alastuey A, Diaz J, et al. Saharan dust, particulate matter and cause-specific mortality: a case-crossover study in Barcelona (Spain). Environ Int. 2012;48:150-5.

58. Rabczenko D, Wojtyniak B, Kuchcik M, Szymalski W, Seroka W, Zmudzka E. Association between high temperature and mortality of Warsaw inhabitants, 2008-2013. Przegl Epidemiol. 2016;70(4):629-40.

59. Ragettli MS, Vicedo-Cabrera AM, Schindler C, Roosli M. Exploring the association between heat and mortality in Switzerland between 1995 and 2013. Environ Res. 2017;158:703-9.

60. Renzi M, Forastiere F, Calzolari R, Cernigliaro A, Madonia G, Michelozzi P, et al. Short-term effects of desert and non-desert PM10 on mortality in Sicily, Italy. Environment International. 2018;120:472-9.

61. Renzi M, Stafoggia M, Faustini A, Cesaroni G, Cattani G, Forastiere F. Analysis of Temporal Variability in the Short-term Effects of Ambient Air Pollutants on Nonaccidental Mortality in Rome, Italy (1998-2014). Environ Health Perspect. 2017;125(6):067019.

62. Samoli E, Stafoggia M, Rodopoulou S, Ostro B, Alessandrini E, Basagana X, et al. Which specific causes of death are associated with short term exposure to fine and coarse particles in Southern Europe? Results from the MED-PARTICLES project. Environ Int. 2014;67:54-61.

63. Salvador C, Nieto R, Linares C, Diaz J, Gimeno L. Effects on daily mortality of droughts in Galicia (NW Spain) from 1983 to 2013. Science of the Total Environment. 2019;662:121-33.

64. Rodopoulou S, Samoli E, Analitis A, Atkinson RW, de'Donato FK, Katsouyanni K. Searching for the best modeling specification for assessing the effects of temperature and humidity on health: a time series analysis in three European cities. Int J Biometeorol. 2015;59(11):1585-96.

65. Samoli E, Stafoggia M, Rodopoulou S, Ostro B, Declercq C, Alessandrini E, et al. Associations between Fine and Coarse Particles and Mortality in Mediterranean Cities: Results from the MED-PARTICLES Project. Environmental Health Perspectives. 2013;121(8):932-8.

66. Schumann B, Edvinsson S, Evengard B, Rocklov J. The influence of seasonal climate variability on mortality in pre-industrial Sweden. Glob Health Action. 2013;6:20153.

67. Scortichini M, de'Donato F, De Sario M, Leone M, Astrom C, Ballester F, et al. The inter-annual variability of heat-related mortality in nine European cities (1990-2010). Environ Health. 2018;17(1):66.

68. Stanisic Stojic S, Stanisic N, Stojic A. Temperature-related mortality estimates after accounting for the cumulative effects of air pollution in an urban area. Environ Health. 2016;15(1):73.

69. Tobias A, Armstrong B, Gasparrini A, Diaz J. Effects of high summer temperatures on mortality in 50 Spanish cities. Environ Health. 2014;13(1):48.

70. Stafoggia M, Schneider A, Cyrys J, Samoli E, Andersen ZJ, Bedada GB, et al. Association Between Short-term Exposure to Ultrafine Particles and Mortality in Eight European Urban Areas. Epidemiology. 2017;28(2):172-80.

71. Scortichini M, De Sario M, de'Donato FK, Davoli M, Michelozzi P, Stafoggia M. Short-Term Effects of Heat on Mortality and Effect Modification by Air Pollution in 25 Italian Cities. Int J Environ Res Public Health. 2018;15(8).

72. Stojic SS, Stanisic N, Stojic A, Sostaric A. Single and combined effects of air pollutants on circulatory and respiratory system-related mortality in Belgrade, Serbia. Journal of Toxicology and Environmental Health-Part a-Current Issues. 2016;79(1):17-27.

73. Willers SM, Jonker MF, Klok L, Keuken MP, Odink J, van den Elshout S, et al. High resolution exposure modelling of heat and air pollution and the impact on mortality. Environ Int. 2016;89-90:102-9.

74. Williams ML, Atkinson RW, Anderson HR, Kelly FJ. Associations between daily mortality in London and combined oxidant capacity, ozone and nitrogen dioxide. Air Quality Atmosphere and Health. 2014;7(4):407-14.

75. Zafeiratou S, Analitis A, Founda D, Giannakopoulos C, Varotsos KV, Sismanidis P, et al. Spatial Variability in the Effect of High Ambient Temperature on Mortality: An Analysis at Municipality Level within the Greater Athens Area. International Journal of Environmental Research and Public Health. 2019;16(19).

76. Xu Y, Dadvand P, Barrera-Gomez J, Sartini C, Mari-Dell'Olmo M, Borrell C, et al. Differences on the effect of heat waves on mortality by sociodemographic and urban landscape characteristics. J Epidemiol Community Health. 2013;67(6):519-25.

77. Zeka A, Browne S, McAvoy H, Goodman P. The association of cold weather and all-cause and cause-specific mortality in the island of Ireland between 1984 and 2007. Environ Health. 2014;13:104.

78. Zhang YQ, Peng MJ, Wang L, Yu CH. Association of diurnal temperature range with daily mortality in England and Wales: A nationwide time-series study. Science of the Total Environment. 2018;619:291-300.

79. Zhang YQ, Yu Y, Peng MJ, Meng RT, Hu KJ, Yu CH. Temporal and seasonal variations of mortality burden associated with hourly temperature variability: A nationwide investigation in England and Wales. Environment International. 2018;115:325-33.

80. Callaly E, Mikulich O, Silke B. Increased winter mortality: the effect of season, temperature and deprivation in the acutely ill medical patient. Eur J Intern Med. 2013;24(6):546-51.

81. Lyons J, Chotirmall SH, O'Riordan D, Silke B. Air quality impacts mortality in acute medical admissions. Qjm. 2014;107(5):347-53.

82. Litchfield IJ, Ayres JG, Jaakkola JJK, Mohammed NI. Is ambient air pollution associated with onset of sudden infant death syndrome: a case-crossover study in the UK. Bmj Open. 2018;8(4).

83. McAllister DA, Morling JR, Fischbacher CM, MacNee W, Wild SH. Socioeconomic deprivation increases the effect of winter on admissions to hospital with COPD: retrospective analysis of 10 years of national hospitalisation data. Prim Care Respir J. 2013;22(3):296-9.

84. Medici CR, Vestergaard CH, Hadzi-Pavlovic D, Munk-Jorgensen P, Parker G. Seasonal variations in hospital admissions for mania: Examining for associations with weather variables over time. J Affect Disord. 2016;205:81-6.

85. Mekontso Dessap A, Contou D, Dandine-Roulland C, Hemery F, Habibi A, Charles-Nelson A, et al. Environmental influences on daily emergency admissions in sickle-cell disease patients. Medicine (Baltimore). 2014;93(29):e280.

86. Abrignani MG, Corrao S, Biondo GB, Lombardo RM, Di Girolamo P, Braschi A, et al. Effects of ambient temperature, humidity, and other meteorological variables on hospital admissions for angina pectoris. Eur J Prev Cardiol. 2012;19(3):342-8.

87. Almeida SM, Silva AV, Sarmento S. Effects of exposure to particles and ozone on hospital admissions for cardiorespiratory diseases in SetuBal, Portugal. J Toxicol Environ Health A. 2014;77(14-16):837-48.

88. Angelici L, Piola M, Cavalleri T, Randi G, Cortini F, Bergamaschi R, et al. Effects of particulate matter exposure on multiple sclerosis hospital admission in Lombardy region, Italy. Environ Res. 2016;145:68-73.

89. Bhaskaran K, Armstrong B, Hajat S, Haines A, Wilkinson P, Smeeth L. Heat and risk of myocardial infarction: hourly level case-crossover analysis of MINAP database. British Medical Journal. 2012;345.

90. Bijelovic S, Dragic N, Bijelovic M, Kovacevic M, Jevtic M, Ninkovic Mrdenovacki O. Impact of climate conditions on hospital admissions for subcategories of cardiovascular diseases. Med Pr. 2017;68(2):189-97.

91. Canova C, Dunster C, Kelly FJ, Minelli C, Shah PL, Caneja C, et al. PM10-induced hospital admissions for asthma and chronic obstructive pulmonary disease: the modifying effect of individual characteristics. Epidemiology. 2012;23(4):607-15.

92. Colais P, Faustini A, Stafoggia M, Berti G, Bisanti L, Cadum E, et al. Particulate air pollution and hospital admissions for cardiac diseases in potentially sensitive subgroups. Epidemiology. 2012;23(3):473-81.

93. Collart P, Dubourg D, Leveque A, Sierra NB, Coppieters Y. Short-term effects of nitrogen dioxide on hospital admissions for cardiovascular disease in Wallonia, Belgium. Int J Cardiol. 2018;255:231-6.

94. de Kluizenaar Y, van Lenthe FJ, Visschedijk AJ, Zandveld PY, Miedema HM, Mackenbach JP. Road traffic noise, air pollution components and cardiovascular events. Noise Health. 2013;15(67):388-97.

95. Lanzinger S, Schneider A, Breitner S, Stafoggia M, Erzen I, Dostal M, et al. Ultrafine and Fine Particles and Hospital Admissions in Central Europe Results from the UFIREG Study. American Journal of Respiratory and Critical Care Medicine. 2016;194(10):1233-41.

96. Lokys HL, Junk J, Krein A. Short-term effects of air quality and thermal stress on non-accidental morbidity-a multivariate meta-analysis comparing indices to single measures. Int J Biometeorol. 2018;62(1):17-27.

97. McWilliams S, Kinsella A, O'Callaghan E. The effects of daily weather variables on psychosis admissions to psychiatric hospitals. Int J Biometeorol. 2013;57(4):497-508.

98. McWilliams S, Kinsella A, O'Callaghan E. Daily weather variables and affective disorder admissions to psychiatric hospitals. Int J Biometeorol. 2014;58(10):2045-57.

99. Ponjoan A, Blanch J, Alves-Cabratosa L, Marti-Lluch R, Comas-Cufi M, Parramon D, et al. Effects of extreme temperatures on cardiovascular emergency hospitalizations in a Mediterranean region: a self-controlled case series study. Environmental Health. 2017;16.

100. Roislien J, Sovik S, Eken T. Seasonality in trauma admissions - Are daylight and weather variables better predictors than general cyclic effects? PLoS One. 2018;13(2):e0192568.

101. Roye D, Zarrabeitia MT, Fdez-Arroyabe P, Alvarez Gutierrez A, Santurtun A. Role of Apparent Temperature and Air Pollutants in Hospital Admissions for Acute Myocardial Infarction in the North of Spain. Rev Esp Cardiol (Engl Ed). 2019;72(8):634-40.

102. Samoli E, Atkinson RW, Analitis A, Fuller GW, Green DC, Mudway I, et al. Associations of short-term exposure to traffic-related air pollution with cardiovascular and respiratory hospital admissions in London, UK. Occupational and Environmental Medicine. 2016;73(5):300-7.

103. Santurtun A, Ruiz PB, Lopez-Delgado L, Sanchez-Lorenzo A, Riancho J, Zarrabeitia MT. Stroke: Temporal Trends and Association with Atmospheric Variables and Air Pollutants in Northern Spain. Cardiovasc Toxicol. 2017;17(3):360-7.

104. Stafoggia M, Samoli E, Alessandrini E, Cadum E, Ostro B, Berti G, et al. Short-term Associations between Fine and Coarse Particulate Matter and Hospitalizations in Southern Europe: Results from the MED-PARTICLES Project. Environmental Health Perspectives. 2013;121(9):1026-33.

105. Tomaskova H, Tomasek I, Slachtova H, Polaufova P, Splichalova A, Michalik J, et al. PM10 AIR POLLUTION AND ACUTE HOSPITAL ADMISSIONS FOR CARDIOVASCULAR AND RESPIRATORY CAUSES IN OSTRAVA. Central European Journal of Public Health. 2016;24:S33-S9.

106. van Loenhout JAF, Delbiso TD, Kiriliouk A, Rodriguez-Llanes JM, Segers J, Guha-Sapir D. Heat and emergency room admissions in the Netherlands. BMC Public Health. 2018;18(1):108.

107. Wichmann J, Rosengren A, Sjoberg K, Barregard L, Sallsten G. Association between ambient temperature and acute myocardial infarction hospitalisations in Gothenburg, Sweden: 1985-2010. PLoS One. 2013;8(4):e62059.

108. Zielinski M, Gasior M, Jastrzebski D, Desperak A, Ziora D. Influence of particulate matter air pollution on exacerbation of chronic obstructive pulmonary disease depending on aerodynamic diameter and the time of exposure in the selected population with coexistent cardiovascular diseases. Adv Respir Med. 2018;86(5):227-33.

109. Dominiak M, Swiecicki L, Rybakowski J. Psychiatric hospitalizations for affective disorders in Warsaw, Poland: Effect of season and intensity of sunlight. Psychiatry Res. 2015;229(1-2):287-94.

110. Ghirardi L, Bisoffi G, Mirandola R, Ricci G, Baccini M. The Impact of Heat on an Emergency Department in Italy: Attributable Visits among Children, Adults, and the Elderly during the Warm Season. Plos One. 2015;10(10).

111. Janke K. Air pollution, avoidance behaviour and children's respiratory health: Evidence from England. Journal of Health Economics. 2014;38:23-42.

112. Piel FB, Tewari S, Brousse V, Analitis A, Font A, Menzel S, et al. Associations between environmental factors and hospital admissions for sickle cell disease. Haematologica. 2017;102(4):666-75.
