# Supplementary material for: The effect of air-pollution and weather exposure on mortality and hospital admission and implications for further research: A systematic scoping review
Source: PLoS One. 2020 Oct 29;15(10):e0241415. doi: 10.1371/journal.pone.0241415 (PMC7595412; doi:10.1371/journal.pone.0241415)
Supplement: S3 Table — (DOCX) [file pone.0241415.s004.docx]

**Title:** The Effect of Air-pollution and weather exposures on Mortality and Hospital Admission and implications for further research: A Systematic Scoping Review

**S3 Table**

S3 Table demonstrate the included article’s reported associations in terms of coefficients with 95% confidence intervals between air pollution and/or weather exposures and mortality and/or hospital admission outcomes.

**The effect of PM10 air pollutant on mortality and hospital admission**

| Outcome | Study | Study design | Lags considered for exposure | RR/IRR/OR/HR/%increase (95%CI or 90%CI or P-value) |
| --- | --- | --- | --- | --- |
| All-cause hospital admission | (Sanyal et al., 2018) | Time-series | NA | RR=1.099 (95%CI=1.072, 1.128)* |
| All-cause mortality | (Carugno et al., 2016) | Time-series | 0-1 days | %increase=0.30 (90%CI=-0.21, 0.70) |
|  | (Stafoggia et al., 2016) | Case-crossover | 0-1 days | %increase=0.51 (95% CI=0.27, 0.75)* |
|  | (Beelen et al., 2014) | Cohort | NA | HR=1.04 (95%CI=1.00, 1.09) |
|  | (Tonne et al., 2016) | Cohort | NA | HR=1.04 (95%CI=0.97, 1.12) |
|  | (Basagana et al., 2015) | Case-crossover | 0 days | %increase=-0.530 (95%CI=-1.618, 0.568) |
|  | (Sanyal et al., 2018) | Time-series | NA | RR=1.029 (95%CI=1.027, 1.031)* |
|  | (Carey et al., 2013) | Cohort | NA | HR=1.04 (95%CI=1.02,1.06)* |
|  | (Hvidtfeldt et al., 2019) | Cohort | NA | HR= 1.15 (95%CI=1.06, 1.25)* |
|  | (Tonne & Wilkinson, 2013) | Cohort | NA | HR=1.01 (95%CI=0.92, 1.10) |
|  | (Alessandrini et al., 2016) | Case-crossover | NA | %increase=0.74 (95%CI= 0.00, 1.49) |
|  | (Burkart et al., 2013) | Time-series | 0-1 days | %increase=0.03 (95%CI=0.01, 0.06)* |
|  | (Burkart et al., 2013) | Time-series | 0-13 days | %increase=0.12 (95%CI=0.07, 0.17)* |
|  | (Faustini et al., 2016) | Case-crossover | 0-1 days | %increase=0.82 (95%CI=0.23, 1.41)* |
|  | (Lanzinger et al., 2016) | Time-series | 0-5 days | %increase=0.8 (95%CI=−3.6, 5.3) |
|  | (Olstrup et al., 2019) | Time-series | 0-2 days | %increase=0.75 (95%CI=0.0, 1.5) |
|  | (Samoli et al., 2013) | Time-series | 0-5 days | %increase=0.28 (95%CI=−0.14, 0.71) |
|  | (Stojic et al., 2016) | Time-series | 0-90 days | %increase=6.02 (95%CI=2.00, 9.04)* |
|  | (Willers et al., 2016) | Case-crossover | 2 days | %increase=1.2 (95%CI=0.2, 2.1)* |
|  | (Nieuwenhuijsen et al., 2018) | Cohort | NA | HR=1.00 (95%CI=0.97,1.03) |
|  | (Badaloni et al., 2017) | Cohort | NA | HR=1.03 (95%CI=1.01, 1.04)* |
|  | (Fischer et al., 2015) | Cohort | NA | HR=1.08 (95% CI=1.07, 1.09)* |
|  | (Pascal et al., 2014) | Time-series | 0-1 days | %increase=0.8 (95%CI=0.2, 1.5)* |
|  | (Renzi et al., 2018) | Time-series | 0-5 days | %increase= 2.27 (95%CI=1.41, 3.14)* |
|  | (Renzi et al., 2017) | Time-series | 0-5 days | %increase= 1.46 (95%CI=0.95, 1.96)* |
|  | (Analitis et al., 2018) | Time-series | 0-3 days | %increase=2.30 (95%CI=1.56, 3.05)* |
|  | (Analitis et al., 2018) | Time-series | 0-6 days | %increase=0.76 (95%CI=0.58, 0.94)* |
|  | (Lyons, Chotirmall, O'Riordan, & Silke, 2014) | Cohort | NA | OR=1.09 (95%CI=0.94, 1.26) |
| Cardiovascular hospital admission | (Colais et al., 2012) | Case-crossover | 0 days | %increase=1.03 (95%CI=0.69, 1.38)* |
|  | (Carugno et al., 2016) | Time-series | 0-3 days | %increase=0.14 (90%CI= -0.31, 0.56) |
|  | (Stafoggia et al., 2016) | Case-crossover | 0-1 days | %increase=0.29 (95% CI= 0.00, 0.58) |
|  | (Basagana et al., 2015) | Case-crossover | 0 days | %increase=1.617 (95%CI=0.624, 2.621)* |
|  | (Lanzinger et al., 2016) | Time-series | 0-5 days | %increase=0.8 (95%CI=−1.9, 3.5) |
|  | (Stafoggia et al., 2013) | Time-series | 0-5 days | %increase=0.30 (95%CI=-0.14, 0.74) |
|  | (Tomaskova et al., 2016) | Time-series | 0 days | %increase=0.01 (95%CI=−0.78, 0.80) |
|  | (Sanyal et al., 2018) | Time-series | NA | RR=1.097 (95%CI=0.899, 1.339) |
| Acute coronary syndrome hospital admission | (Colais et al., 2012) | Case-crossover | 0-1 days | %increase=1.13 (95%CI=0.37, 1.89)* |
| Acute myocardial infarction hospital admission | (Roye et al., 2019) | Time-series | 0 days | RR=1.02 (95%CI=1.00, 1.04) |
|  | (Tonne et al., 2016) | Cohort | NA | HR=1.05 (95%CI=0.98, 1.12) |
| Arrhythmias and conduction disorders hospital admission | (Colais et al., 2012) | Case-crossover | 0 days | %increase=1.00 (95%CI=0.22, 1.78)* |
| Heart failure hospital admission | (Colais et al., 2012) | Case-crossover | 0 days | %increase=1.37 (95%CI=0.74, 2.00)* |
| Stroke hospital admission | (Roye et al., 2019) | Time-series | 0-14 days | RR=0.99 (95%CI=0.97, 1.00) |
| Pulmonary embolism hospital admission | (de Miguel-Diez et al., 2016) | Case-crossover | NA | OR=1.04 (95%CI=1.02, 1.05)* |
| Cardiovascular mortality | (Sanyal et al., 2018) | Time-series | NA | RR=1.047 (95%CI=1.045, 1.051)* |
|  | (Carey et al., 2013) | Cohort | NA | HR=1.02 (95%CI=0.99,1.04) |
|  | (Fischer et al., 2015) | Cohort | NA | HR =1.06 (95% CI=1.04, 1.08)* |
|  | (Stojic et al., 2016) | Time-series | 0-90 days | %increase=4.01 (95%CI=–1.00, 9.04) |
|  | (Faustini et al., 2016) | Case-crossover | 0-1 days | %increase=0.94 (95%CI=0.21,1.67)* |
|  | (Samoli et al., 2014) | Time-series | 0-5 days | %increase=0.79 (95%CI=0.08, 1.50)* |
|  | (Carugno et al., 2016) | Time-series | 0-1 days | %increase=0.30 (90%CI= -0.21, 0.82) |
|  | (Stafoggia et al., 2016) | Case-crossover | 0-5 days | %increase=0.66 (95% CI=–0.02, 1.34) |
|  | (Basagana et al., 2015) | Case-crossover | 0 days | %increase=-1.325 (95%CI=-3.101, 0.482) |
|  | (Badaloni et al., 2017) | Cohort | NA | HR=1.04 (95%CI=1.01, 1.07)* |
|  | (Hvidtfeldt et al., 2019) | Cohort | NA | HR=1.34 (95%CI=1.14, 1.57)* |
|  | (Pascal et al., 2014) | Time-series | 0-1 days | %increase=0.9 (95%CI=0.2, 1.7)* |
|  | (Perez et al., 2012) | Case-crossover | 1 day | OR=1.041 (95%CI=1.018, 1.066)* |
|  | (Renzi et al., 2018) | Time-series | 0-5 days | %increase=2.38 (95%CI=1.35, 3.43)* |
|  | (Samoli et al., 2013) | Time-series | 0-5 days | %increase=0.54 (95%CI=0.09, 0.99)* |
|  | (Hansell et al., 2016) | Cohort | NA | OR=1.12 (95% CI= 1.01, 1.25)* |
|  | (Lanzinger et al., 2016) | Time-series | 0-5 days | %increase=2.3 (95%CI=−2.9, 7.8) |
|  | (Beelen et al., 2014) | Cohort | NA | HR=1.02 (95%CI=0.92, 1.14) |
| Myocardial infarction mortality | (Beelen et al., 2014) | Cohort | NA | HR=0.94 (95%CI=0.81,1.09) |
| Ischemic heart disease mortality | (Badaloni et al., 2017) | Cohort | NA | HR=1.05 (95%CI=1.00, 1.09) |
|  | (Beelen et al., 2014) | Cohort | NA | HR=0.93 (95%CI=0.77,1.13) |
|  | (Pascal et al., 2014) | Time-series | 0-1 days | %increase=0.8 (95%CI=-0.4, 2.0) |
| Stroke mortality | (Roye et al., 2019) | Time-series | 0-14 days | RR=1.02 (95%CI=0.99, 1.05) |
| Pulmonary embolism mortality | (de Miguel-Diez et al., 2016) | Case-crossover | NA | OR=1.02 (95%CI=0.96, 1.07) |
| Cerebrovascular hospital admission | (Carugno et al., 2016) | Time-series | 0-3 days | %increase=0.54 (90%CI= -0.14, 1.23) |
|  | (de Kluizenaar et al., 2013) | Cohort | NA | RR=1.06 (95%CI=1.01, 1.11)* |
| Cerebrovascular mortality | (Beelen et al., 2014) | Cohort | NA | HR=1.22 (95%CI=0.91,1.63) |
|  | (Pascal et al., 2014) | Time-series | 0-1 days | %increase=1.5 (95%CI=-0.1, 3.1) |
|  | (Perez et al., 2012) | Case-crossover | 1 day | OR=1.039 (95%CI=0.993, 1.087) |
|  | (Samoli et al., 2014) | Time-series | 0-5 days | %increase=0.06 (95%CI=−0.86, 0.99) |
| COPD hospital admission | (Canova et al., 2012) | Case-crossover | 0-3 days | OR=1.35 (95%CI=1.04, 1.76)* |
|  | (Zielinski et al.,2018) | Time-series | 90 days | RR=1.00 (P-value=0.85) |
| COPD mortality | (Samoli et al., 2014) | Time-series | 0-5 days | %increase=1.15 (95%CI=−0.57, 2.90) |
|  | (Faustini et al., 2016) | Case-crossover | 0-1 days | %increase=1.39 (95%CI= 0.36, 2.43)* |
| Diabetes hospital admission | (Lanzinger et al., 2016) | Time-series | 0-5 days | %increase=3.9 (95%CI=−1.7, 9.8) |
| Diabetes mortality | (Samoli et al., 2014) | Time-series | 0-5 days | %increase=0.93 (95%CI=−1.88, 3.82) |
| Multiple sclerosis hospital admission | (Angelici et al., 2016) | Time-series | 0-1 days | RR=1.33 (95%CI=1.23, 1.43)* |
|  | (Angelici et al., 2016) | Time-series | 0-21 days | RR=1.15 (95%CI=1.14, 1.16)* |
| Respiratory hospital admission | (Carugno et al., 2016) | Time-series | 0-3 days | %increase=0.77 (90%CI= 0.31, 1.32)* |
|  | (Stafoggia et al., 2016) | Case-crossover | 0-5 days | %increase=0.69 (95% CI=0.20, 1.19)* |
|  | (Basagana et al., 2015) | Case-crossover | 0 days | %increase=1.710 (95%CI=-0.854, 4.342) |
|  | (Sanyal et al., 2018) | Time-series | NA | RR=1.181 (95%CI=0.970, 1.439) |
|  | (Almeida, Silva, & Sarmento, 2014) | Time-series | NA | %increase=0.9 (P-value=0.008)* |
|  | (Lanzinger et al., 2016) | Time-series | 0-5 days | %increase=7.3 (95%CI=4.4, 10.3)* |
|  | (Stafoggia et al., 2013) | Time-series | 0-5 days | %increase=1.15 (95%CI=0.21, 2.11)* |
|  | (Tomaskova et al., 2016) | Time-series | 0 days | %increase=1.52 (95%CI=0.42, 2.63)* |
| Respiratory mortality | (Carugno et al., 2016) | Time-series | 0-1 days | % change=1.64 (90%CI= 0.56, 2.72)* |
|  | (Stafoggia et al., 2016) | Case-crossover | 0-5 days | %increase=2.01 (95% CI=0.92, 3.12)* |
|  | (Basagana et al., 2015) | Case-crossover | 0 days | %increase=1.027 (95%CI=-2.603, 4.793) |
|  | (Sanyal et al., 2018) | Time-series | NA | RR=1.056 (95%CI=1.043, 1.069)* |
|  | (Carey et al., 2013) | Cohort | NA | HR=1.11 (95%CI=1.08,1.15)* |
|  | (Dimakopoulou et al., 2014) | Cohort | NA | HR= 0.86 (95%CI=0.67, 1.04) |
|  | (Fischer et al., 2015) | Cohort | NA | HR = 1.13 (95% CI=1.10, 1.17)* |
|  | (Hansell et al., 2016) | Cohort | NA | OR=1.22 (95% CI =1.04, 1.44)* |
|  | (Pascal et al., 2014) | Time-series | 0-1 days | %increase=-0.5 (95%CI=-1.9, 0.9) |
|  | (Perez et al., 2012) | Case-crossover | 2 days | OR=1.053 (95%CI=1.014, 1.094)* |
|  | (Renzi et al., 2018) | Time-series | 0-5 days | %increase=8.15 (95%CI=6.82, 9.50)* |
|  | (Samoli et al., 2013) | Time-series | 0-5 days | %increase=1.12 (95%CI=0.29, 1.95)* |
|  | (Stojic et al., 2016) | Time-series | 0-90 days | %increase=9.04 (95%CI=–7.97, 25.32) |
|  | (Hvidtfeldt et al., 2019) | Cohort | NA | HR=1.08 (95%CI=0.91, 1.29) |
|  | (Lanzinger et al., 2016) | Time-series | 0-5 days | %increase=-5.1 (95%CI=−12.6, 3.1) |
| Lower respiratory tract infection mortality | (Samoli et al., 2014) | Time-series | 0-5 days | %increase=0.80 (95%CI=−1.10, 2.75) |
| Lung cancer mortality | (Carey et al., 2013) | Cohort | NA | HR=1.03 (95%CI=0.98,1.08) |
|  | (Fischer et al., 2015) | Cohort | NA | HR =1.26 (95% CI=1.21, 1.30)* |
| Sudden infant death | (Litchfield, Ayres, Jaakkola, & Mohammed, 2018) | Case-crossover | 1 day | OR=1.15 (95%CI=1.05, 1.28)* |

*Significant result based on the 95% confidence interval or the P-value (<0.05)

**The effect of PM2.5 air pollutant on mortality and hospital admission**

| Outcome | Study | Study design | Lags considered for exposure | RR/IRR/OR/HR/%increase (95%CI or 90%CI or P-value) |
| --- | --- | --- | --- | --- |
| All-cause hospital admission | (Sanyal et al., 2018) | Time-series | NA | RR=1.107 (95%CI=1.079, 1.136)* |
| All-cause mortality | (Beelen et al., 2014) | Cohort | NA | HR=1.07 (95%CI=1.02, 1.13)* |
|  | (Tonne et al., 2016) | Cohort | NA | HR=1.04 (95%CI=0.95, 1.14) |
|  | (Basagana et al., 2015) | Case-crossover | 0 days | %increase=0.178 (95%CI=-1.450, 1.833) |
|  | (Sanyal et al., 2018) | Time-series | NA | RR=1.024 (95%CI=1.022, 1.026)* |
|  | (Carey et al., 2013) | Cohort | NA | HR=1.04 (95%CI=1.02,1.06)* |
|  | (Hvidtfeldt et al., 2019) | Cohort | NA | HR=1.16 (95%CI=1.08, 1.24)* |
|  | (Tonne & Wilkinson, 2013) | Cohort | NA | HR=1.20 (95%CI=1.04, 1.38)* |
|  | (Alessandrini et al., 2016) | Case-crossover | NA | %increase=1.05 (95%CI= 0.25, 1.86)* |
|  | (Lanzinger et al., 2016) | Time-series | 0-5 days | %increase=0.3 (95%CI=−3.7, 4.5) |
|  | (Meister, Johansson, & Forsberg, 2012) | Time-series | 0-1 days | %increase=1.46 (95%CI=0.07, 2.84)* |
|  | (Olstrup et al., 2019) | Time-series | 0-2 days | %increase=0.00 (95%CI=-0.75, 0.75) |
|  | (Samoli et al., 2013) | Time-series | 0-5 days | %increase=0.70 (95%CI=0.22, 1.18)* |
|  | (Nieuwenhuijsen et al., 2018) | Cohort | NA | HR=1.03 (95%CI=0.99,1.06) |
|  | (Badaloni et al., 2017) | Cohort | NA | HR=1.03 (95%CI=1.01, 1.05)* |
|  | (Madsen et al., 2012) | Case-crossover | 0-5 days | %increase=2.8 (95%CI=1.2, 4.4)* |
|  | (Pascal et al., 2014) | Time-series | 0-1 days | %increase= 0.7 (95%CI=-0.1, 1.6) |
|  | (Renzi et al., 2017) | Time-series | 0-5 days | increase= 1.75 (95%CI=0.87, 2.64)* |
|  | (Cesaroni et al., 2013) | Cohort | NA | HR=1.02 (95%CI=1.02, 1.03)* |
| Cardiovascular hospital admission | (Basagana et al., 2015) | Case-crossover | 0 days | %increase=1.887 (95%CI=0.508, 3.284)* |
|  | (Lanzinger et al., 2016) | Time-series | 0-5 days | %increase=1.7 (95%CI=−0.1, 3.6) |
|  | (Stafoggia et al., 2013) | Time-series | 0-5 days | %increase=0.49 (95%CI=0.03, 0.95)* |
|  | (Sanyal et al., 2018) | Time-series | NA | RR=1.225 (95%CI=0.967, 1.551) |
| Cardiovascular mortality | (Sanyal et al., 2018) | Time-series | NA | RR=1.022 (95%CI=1.015, 1.029)* |
|  | (Carey et al., 2013) | Cohort | NA | HR=1.02 (95%CI=1.00,1.05) |
|  | (Madsen et al., 2012) | Case-crossover | 0-5 days | %increase=2.7 (95%CI=0.3, 5.0)* |
|  | (Samoli et al., 2014) | Time-series | 0-5 days | %increase=1.33 (95%CI=0.27, 2.40)* |
|  | (Basagana et al., 2015) | Case-crossover | 0 days | %increase=-1.354 (95%CI=-4.673, 2.080) |
|  | (Badaloni et al., 2017) | Cohort | NA | HR=1.05 (95%CI=1.02, 1.08)* |
|  | (Hvidtfeldt et al., 2019) | Cohort | NA | HR=1.32 (95%CI=1.16, 1.50)* |
|  | (Pascal et al., 2014) | Time-series | 0-1 days | %increase= 0.7 (95%CI=-0.2, 1.6) |
|  | (Perez et al., 2012) | Case-crossover | 1 day | OR=1.055 (95%CI=1.023, 1.089)* |
|  | (Samoli et al., 2013) | Time-series | 0-5 days | %increase=0.86 (95%CI=0.15, 1.57)* |
|  | (Cesaroni et al., 2013) | Cohort | NA | HR=1.04 (95%CI=1.03, 1.05)* |
|  | (Lanzinger et al., 2016) | Time-series | 0-5 days | %increase=1.6 (95%CI=−2.8, 6.2) |
|  | (Beelen et al., 2014) | Cohort | NA | HR= 0.99 (95%CI=0.91,1.08) |
| Cerebrovascular mortality | (Beelen et al., 2014) | Cohort | NA | HR=1.21 (95%CI=0.87,1.69) |
|  | (Cesaroni et al., 2013) | Cohort | NA | HR=1.01 (95%CI=0.99, 1.05) |
|  | (Pascal et al., 2014) | Time-series | 0-1 days | %increase= 1.5 (95%CI=-0.9, 4.0) |
|  | (Perez et al., 2012) | Case-crossover | 1 day | OR=1.049 (95%CI=0.987, 1.116) |
|  | (Samoli et al., 2014) | Time-series | 0-5 days | %increase=0.78 (95%CI=−0.86, 2.45) |
| COPD hospital admission | (Zielinski et al., 2018) | Time-series |  | RR=1.32 (P-value <0.0001)* |
| COPD mortality | (Samoli et al., 2014) | Time-series | 0-5 days | %increase=2.53 (95%CI=−0.01, 5.14) |
| Diabetes hospital admission | (Lanzinger et al., 2016) | Time-series | 0-5 days | %increase=4.1 (95%CI=−1.5, 9.9) |
| Diabetes mortality | (Samoli et al., 2014) | Time-series | 0-5 days | %increase=2.02 (95%CI=−1.51, 5.68) |
| Ischemic heart disease mortality | (Badaloni et al., 2017) | Cohort | NA | HR=1.06 (95%CI=1.01, 1.11)* |
|  | (Beelen et al., 2014) | Cohort | NA | HR=0.98 (95%CI=0.74,1.30) |
|  | (Cesaroni et al., 2013) | Cohort | NA | HR=1.06 (95%CI=1.04, 1.07)* |
|  | (Pascal et al., 2014) | Time-series | 0-1 days | %increase= 0.6 (95%CI=-1.1, 2.3) |
| Lower respiratory tract infection mortality | (Samoli et al., 2014) | Time-series | 0-5 days | %increase=1.37 (95%CI=−1.94, 4.78) |
| Lung cancer mortality | (Carey et al., 2013) | Cohort | NA | HR=1.04 (95%CI=0.99,1.09) |
|  | (Cesaroni et al., 2013) | Cohort | NA | HR=1.03 (95%CI=1.01, 1.06)* |
| Myocardial infarction hospital readmission | (Tonne et al., 2016) | Cohort | NA | HR=1.05 (95%CI=0.97, 1.15) |
| Myocardial infarction mortality | (Beelen et al., 2014) | Cohort | NA | HR=0.96 (95%CI=0.70,1.32) |
| Respiratory hospital admission | (Basagana et al., 2015) | Case-crossover | 0 days | %increase=0.735 (95%CI=-0.860, 2.358) |
|  | (Sanyal et al., 2018) | Time-series | NA | RR=1.2 (95%CI=0.990, 1.454) |
|  | (Almeida, Silva, & Sarmento, 2014) | Time-series | NA | %increase=0.4 (P-value=0.240) |
|  | (Lanzinger et al., 2016) | Time-series | 0-5 days | %increase=7.5 (95%CI=4.9, 10.2)* |
|  | (Stafoggia et al., 2013) | Time-series | 0-5 days | %increase=1.36 (95%CI=0.23, 2.49)* |
| Respiratory mortality | (Basagana et al., 2015) | Case-crossover | 0 days | %increase=0.987 (95%CI=-4.419, 6.699) |
|  | (Sanyal et al., 2018) | Time-series | NA | RR=1.037 (95%CI=1.029, 1.044)* |
|  | (Carey et al., 2013) | Cohort | NA | HR=1.12 (95%CI=1.08,1.16)* |
|  | (Cesaroni et al., 2013) | Cohort | NA | HR=1.05 (95%CI=1.02, 1.07)* |
|  | (Dimakopoulou et al., 2014) | Cohort | NA | HR= 0.89 (95%CI=0.66, 1.12) |
|  | (Madsen et al., 2012) | Case-crossover | 0-5 days | %increase=3.0 (95%CI=-1.0, 7.1) |
|  | (Pascal et al., 2014) | Time-series | 0-1 days | %increase= -1.1 (95%CI=-3.7, 1.5) |
|  | (Perez et al., 2012) | Case-crossover | 2 days | OR=1.067 (95%CI=1.011, 1.126)* |
|  | (Samoli et al., 2013) | Time-series | 0-5 days | %increase=1.91 (95%CI=0.71, 3.12)* |
|  | (Hvidtfeldt et al., 2019) | Cohort | NA | HR=1.11 (95%CI=0.96, 1.29) |
|  | (Lanzinger et al., 2016) | Time-series | 0-5 days | %increase=-2.4 (95%CI=−10.5, 6.4) |

*Significant result based on the 95% confidence interval or the P-value (<0.05)

**The effect of O3 air pollutant on mortality and hospital admission**

| Outcome | Study | Study design | Lags considered for exposure | RR/IRR/OR/HR/%increase (95%CI or 90%CI or P-value) |
| --- | --- | --- | --- | --- |
| All-cause hospital admission | (Sanyal et al., 2018) | Time-series | NA | RR=0.998 (95%CI=0.963, 1.035) |
| All-cause mortality | (Hunova et al., 2013) | Time-series | 1 day | RR=1.01 (95% CI=0.99,1.02) |
|  | (Tonne et al., 2016) | Cohort | NA | HR=0.98 (95%CI=0.91, 1.05) |
|  | (Sanyal et al., 2018) | Time-series | NA | RR=0.991 (95%CI=0.990, 0.991)* |
|  | (Carey et al., 2013) | Cohort | NA | HR=0.96 (95%CI=0.93,0.98)* |
|  | (Hvidtfeldt et al., 2019) | Cohort | NA | HR=0.89 (95%CI=0.86, 0.93)* |
|  | (Burkart et al., 2013) | Time-series | 0-1 days | %increase=0.04 (95%CI=0.01, 0.07)* |
|  | (Burkart et al., 2013) | Time-series | 0-13 days | %increase=0.11 (95%CI=0.05, 0.17)* |
|  | (Olstrup et al., 2019) | Time-series | 0-2 days | %increase=2.0 (95%CI=1.0, 3.0)* |
|  | (Willers et al., 2016) | Case-crossover | 2 days | %increase=1.4 (95%CI=-0.4, 3.2) |
|  | (Williams et al., 2014) | Time-series | 1 day | %increase=1.3 (95%CI=0.1, 1.9)* |
|  | (Analitis et al., 2018) | Time-series | 0-3 days | %increase=2.20 (95%CI=1.28, 3.13)* |
|  | (Analitis et al., 2018) | Time-series | 0-6 days | %increase=0.75 (95%CI=0.55, 0.95)* |
| Cardiovascular hospital admission | (Hunova et al., 2013) | Time-series | 1 day | RR=0.99 (95% CI=0.98,1.00) |
|  | (Sanyal et al., 2018) | Time-series | NA | RR=0.919 (95%CI=0.700, 1.208) |
| Cardiovascular mortality | (Hunova et al., 2013) | Time-series | 1 day | RR=1.01 (95% CI=0.99,1.02) |
|  | (Hvidtfeldt et al., 2019) | Cohort | NA | HR=0.86 (95%CI=0.79, 0.92)* |
|  | (Sanyal et al., 2018) | Time-series | NA | RR=0.993 (95%CI=0.941, 1.049) |
|  | (Carey et al., 2013) | Cohort | NA | HR=0.96 (95%CI=0.94,0.98)* |
| Ischemic stroke hospital admission | (Roye et al., 2019) | Time-series | 0-14 days | RR=1.01 (95%CI=0.99, 1.02) |
| Ischemic stroke mortality | (Roye et al., 2019) | Time-series | 0-14 days | RR=0.99 (95%CI=0.96, 1.02) |
| Lung cancer mortality | (Carey et al., 2013) | Cohort | NA | HR=0.94 (95%CI=0.90,0.98)* |
| Myocardial infarction hospital readmission | (Tonne et al., 2016) | Cohort | NA | HR=0.96 (95%CI=0.89, 1.03) |
| Pulmonary embolism hospital admission | (de Miguel-Diez et al., 2016) | Case-crossover | NA | OR=0.93 (95%CI=0.91, 0.95)* |
| Pulmonary embolism mortality | (de Miguel-Diez et al., 2016) | Case-crossover | NA | OR=1.00 (95%CI=0.94, 1.07) |
| Respiratory hospital admission | (Hunova et al., 2013) | Time-series | 1 day | RR=1.01 (95% CI=0.99,1.03) |
|  | (Sanyal et al., 2018) | Time-series | NA | RR=0.869 (95%CI=0.645, 1.172) |
|  | (Janke, 2014) | Time-series | 0-4 days | Coefficient=0.026 (95%CI=0.007, 0.104)* |
| Respiratory mortality | (Hunova et al., 2013) | Time-series | 1 day | RR=1.08 (95% CI=1.03,1.14)* |
|  | (Sanyal et al., 2018) | Time-series | NA | RR=1.00 (95%CI=0.999, 1.002) |
|  | (Carey et al., 2013) | Cohort | NA | HR=0.93 (95%CI=0.90,0.96)* |
|  | (Hvidtfeldt et al., 2019) | Cohort | NA | HR=0.91 (95%CI=0.84–0.99)* |
| Sudden infant death | (Litchfield, Ayres, Jaakkola, & Mohammed, 2018) | Case-crossover | 1 day | OR=0.82 (95%CI=0.72, 0.92)* |

*Significant result based on the 95% confidence interval or the P-value (<0.05)

**The effect of NO2 air pollutant on mortality and hospital admission**

| Outcome | Study | Study design | Lags considered for exposure | RR/IRR/OR/HR/%increase (95%CI or 90%CI or P-value) |
| --- | --- | --- | --- | --- |
| All-cause hospital admission | (Sanyal et al., 2018) | Time-series | NA | RR=1.046 (95%CI=1.020, 1.074)* |
| All-cause mortality | (Carugno et al., 2016) | Time-series | 0-1 days | %increase=0.70 (90%CI= 0.20, 1.18)* |
|  | (Beelen et al., 2014) | Cohort | NA | HR=1.01 (95%CI=0.99, 1.03) |
|  | (Tonne et al., 2016) | Cohort | NA | HR=1.04 (95%CI=0.97, 1.10) |
|  | (Sanyal et al., 2018) | Time-series | NA | RR=1.002 (95%CI=1.001, 1.002)* |
|  | (Carey et al., 2013) | Cohort | NA | HR=1.06 (95%CI=1.03,1.08)* |
|  | (Hvidtfeldt et al., 2019) | Cohort | NA | HR=1.09 (95%CI=1.06, 1.12)* |
|  | (Tonne & Wilkinson, 2013) | Cohort | NA | HR=1.01 (95%CI=0.98, 1.04) |
|  | (Atkinson et al., 2016) | Time-series | 1 day | %increase=− 0.43 (95%CI=−1.24, 0.40) |
|  | (Deguen et al., 2015) | Case-crossover | 0-5 days | %increase=0.94 (95%CI =0.08,1.80)* |
|  | (Lanzinger et al., 2016) | Time-series | 0-5 days | %increase=0.4 (95%CI=−3.1, 4.1) |
|  | (Linares et al., 2018) | Time-series | NA | RR=1.012 (95%CI=1.010, 1.014)* |
|  | (Olstrup et al., 2019) | Time-series | 0-2 days | %increase=-1.5 (95%CI=-2.5, -0.5)* |
|  | (Stojic et al., 2016) | Time-series | 0-90 days | %increase=10.05 (95%CI=–1.00, 21.22) |
|  | (Willers et al., 2016) | Case-crossover | 2 days | %increase=1.7 (95%CI=0.4, 3.1)* |
|  | (Nieuwenhuijsen et al., 2018) | Cohort | NA | HR=1.01 (95%CI=1.00,1.02) |
|  | (Raaschou-Nielsen et al., 2012) | Cohort | NA | HR=1.13 (95%CI=1.04, 1.23)* |
|  | (Stockfelt et al., 2015) | Cohort | NA | HR=1.02 (95%CI=1.01, 1.04)* |
|  | (Fischer et al., 2015) | Cohort | NA | HR=1.03 (95% CI=1.02, 1.03)* |
|  | (Madsen et al., 2012) | Case-crossover | 0-5 days | %increase=0.6 (95%CI=-1.1, 2.3) |
|  | (Renzi et al., 2017) | Time-series | 0-5 days | %increase=3.03 (95%CI=2.44, 3.63)* |
|  | (Williams et al., 2014) | Time-series | 1 day | %increase=1.7 (95%CI=0.8, 2.5)* |
|  | (Cesaroni et al., 2013) | Cohort | NA | HR=1.03 (95%CI=1.02, 1.04)* |
|  | (Analitis et al., 2018) | Time-series | 0-3 days | %increase=2.16 (95%CI=1.43, 2.90)* |
|  | (Analitis et al., 2018) | Time-series | 0-6 days | %increase=0.88 (95%CI=0.68, 1.08)* |
|  | (Lyons, Chotirmall, O'Riordan, & Silke, 2014) | Cohort | NA | OR=1.17 (95%CI=1.10, 1.35)* |
| Acute myocardial infarction hospital admission | (Collart et al., 2018) | Time-series | 0 days | %increase=2.8 (95%CI=0.8, 4.9)* |
|  | (Roye et al., 2019) | Time-series | 0 days | RR=1.00 (95%CI=0.97, 1.04) |
| Arrhythmia hospital admission | (Collart et al., 2018) | Time-series | 0 days | %increase=3.7 (95%CI=2.1, 5.3)* |
| Cardiac rhythm disturbances mortality | (Raaschou-Nielsen et al., 2012) | Cohort | NA | HR=1.01 (95%CI=0.28,3.65) |
| Cardiovascular hospital admission | (Sanyal et al., 2018) | Time-series | NA | RR=1.071 (95%CI=0.876, 1.310) |
|  | (Carugno et al., 2016) | Time-series | 0-3 days | %increase=1.14 (90%CI= 0.51, 1.83)* |
|  | (Collart et al., 2018) | Time-series | 0 days | %increase=3.5 (95%CI=2.4, 4.7)* |
|  | (Lanzinger et al., 2016) | Time-series | 0-5 days | %increase=-0.8 (95%CI=−3.5, 2.0) |
|  | (Samoli et al., 2016) | Time-series | 1 day | %increase=0.92 (95%CI=−0.15, 2.00) |
| Cardiovascular mortality | (Sanyal et al., 2018) | Time-series | NA | RR=1.003 (95%CI=1.003, 1.004)* |
|  | (Carey et al., 2013) | Cohort | NA | HR=1.03 (95%CI=1.00,1.07) |
|  | (Fischer et al., 2015) | Cohort | NA | HR=1.00 (95% CI=0.99, 1.01) |
|  | (Linares et al., 2018) | Time-series | NA | RR=1.016 (95%CI=1.012, 1.021)* |
|  | (Madsen et al., 2012) | Case-crossover | 0-5 days | %increase=0.5 (95%CI=-2.0, 3.0) |
|  | (Stojic et al., 2016) | Time-series | 0-90 days | %increase=3.00 (95%CI=–11.93, 19.18) |
|  | (Carugno et al., 2016) | Time-series | 0-1 days | % change=1.12 (90%CI= 0.30, 1.95)* |
|  | (Cesaroni et al., 2013) | Cohort | NA | HR=1.03 (95%CI=1.02, 1.04)* |
|  | (Dehbi et al., 2017) | Cohort | NA | HR=0.97 (95%CI=0.81, 1.16) |
|  | (Hvidtfeldt et al., 2019) | Cohort | NA | HR=1.13 (95%CI=1.07, 1.20)* |
|  | (Raaschou-Nielsen et al., 2012) | Cohort | NA | HR=1.26 (95%CI=1.06, 1.51)* |
|  | (Atkinson et al., 2016) | Time-series | 1 day | %increase=− 1.29 (95%CI=−2.72, 0.17) |
|  | (Stockfelt et al., 2015) | Cohort | NA | HR=1.02 (95%CI=1.00, 1.05) |
|  | (Lanzinger et al., 2016) | Time-series | 0-5 days | %increase=0.8 (95%CI=−3.7, 5.5) |
|  | (Beelen et al., 2014) | Cohort | NA | HR= 1.01 (95%CI=0.97,1.06) |
| Cerebrovascular disease hospital admission | (de Kluizenaar et al., 2013) | Cohort | NA | RR=1.02 (95%CI=1.01, 1.03)* |
|  | (Carugno et al., 2016) | Time-series | 0-3 days | %increase=1.70 (90%CI= 0.60, 2.66)* |
| Cerebrovascular mortality | (Beelen et al., 2014) | Cohort | NA | HR=1.01 (95%CI=0.93,1.10) |
|  | (Cesaroni et al., 2013) | Cohort | NA | HR=1.03 (95%CI=1.00, 1.06) |
|  | (Raaschou-Nielsen et al., 2012) | Cohort | NA | HR=1.11 (95%CI=0.78,1.63) |
|  | (Stockfelt et al., 2015) | Cohort | NA | HR=1.03 (95%CI=0.97, 1.09) |
| Diabetes hospital admission | (Lanzinger et al., 2016) | Time-series | 0-5 days | %increase=-0.8 (95%CI=−9.1, 8.2) |
| Heart failure mortality | (Raaschou-Nielsen et al., 2012) | Cohort | NA | HR=0.94 (95%CI=0.35,2.53) |
| Ischemic heart disease mortality | (Beelen et al., 2014) | Cohort | NA | HR=1.00 (95%CI=0.91,1.09) |
|  | (Cesaroni et al., 2013) | Cohort | NA | HR=1.05 (95%CI=1.03, 1.07)* |
|  | (Raaschou-Nielsen et al., 2012) | Cohort | NA | HR=1.12 (95%CI=0.85, 1.47) |
|  | (Stockfelt et al., 2015) | Cohort | NA | HR=1.02 (95%CI=0.99, 1.05) |
| Ischemic stroke mortality | (Roye et al., 2019) | Time-series | 0-14 days | RR=1.01 (95%CI=0.99, 1.04) |
| Lung cancer mortality | (Carey et al., 2013) | Cohort | NA | HR=1.11 (95%CI=1.05,1.17)* |
|  | (Cesaroni et al., 2013) | Cohort | NA | HR=1.05 (95%CI=1.02, 1.08)* |
|  | (Fischer et al., 2015) | Cohort | NA | HR =1.10 (95% CI= 1.09, 1.11)* |
| Myocardial infarction hospital readmission | (Tonne et al., 2016) | Cohort | NA | HR=1.04 (95%CI=0.98, 1.11) |
| Myocardial infarction mortality | (Beelen et al., 2014) | Cohort | NA | HR=0.98 (95%CI=0.88,1.09) |
|  | (Stockfelt et al., 2015) | Cohort | NA | HR=1.03 (95%CI=0.99, 1.07) |
| Pulmonary embolism hospital admission | (de Miguel-Diez et al., 2016) | Case-crossover | NA | OR=1.11 (95%CI=1.09, 1.13)* |
| Pulmonary embolism mortality | (de Miguel-Diez et al., 2016) | Case-crossover | NA | OR=1.05 (95%CI=1.00, 1.11) |
| Respiratory hospital admission | (Carugno et al., 2016) | Time-series | 0-3 days | %increase=1.20 (90%CI= 0.53, 1.81)* |
|  | (Sanyal et al., 2018) | Time-series | NA | RR=1.17 (95%CI=0.904, 1.513) |
|  | (Lanzinger et al., 2016) | Time-series | 0-5 days | %increase=6.8 (95%CI=−0.2, 14.2) |
|  | (Samoli et al., 2016) | Time-series | 2 days | %increase=-0.60 (95%CI=−1.64, 0.46) |
|  | (Janke, 2014) | Time-series | 0-4 days | Coefficient=0.036 (95%CI=0.008, 0.090)* |
| Respiratory mortality | (Carugno et al., 2016) | Time-series | 0-1 days | % change=0.46 (90%CI= -1.23, 2.18) |
|  | (Sanyal et al., 2018) | Time-series | NA | RR=0.998 (95%CI=0.997, 1.00) |
|  | (Carey et al., 2013) | Cohort | NA | HR=1.15 (95%CI=1.10,1.20)* |
|  | (Cesaroni et al., 2013) | Cohort | NA | HR=1.01 (95%CI=0.99, 1.03) |
|  | (Dimakopoulou et al., 2014) | Cohort | NA | HR= 0.97 (95%CI=0.89, 1.05) |
|  | (Fischer et al., 2015) | Cohort | NA | HR =1.02 (95%CI=1.01, 1.03)* |
|  | (Atkinson et al., 2016) | Time-series | 2 days | %increase=− 0.04 (95%CI=−1.96, 1.91) |
|  | (Linares et al., 2018) | Time-series | NA | RR=1.028 (95%CI=1.020, 1.036)* |
|  | (Madsen et al., 2012) | Case-crossover | 0-5 days | %increase=-0.6 (95%CI=-5.0, 3.9) |
|  | (Hvidtfeldt et al., 2019) | Cohort | NA | HR=1.07 (95%CI=1.01, 1.14)* |
|  | (Stockfelt et al., 2015) | Cohort | NA | HR=1.00 (95%CI=0.93, 1.06) |
|  | (Lanzinger et al., 2016) | Time-series | 0-5 days | %increase=-1.2 (95%CI=−12.8, 11.9) |
|  | (Stojic et al., 2016) | Time-series | 0-90 days | %increase=5.01 (95%CI=–47.82, 60.78) |
| Stroke hospital admission | (Santurtun et al., 2017) | Time-series | NA | %increase=8.3 (P-value = 0.012) |
|  | (Collart et al., 2018) | Time-series | 0 days | %increase=4.5 (95%CI=2.8, 6.3)* |
|  | (Roye et al., 2019) | Time-series | 0-14 days | RR=0.99 (95%CI=0.98, 1.00) |
|  | (Collart et al., 2018) | Time-series | 2 days | %increase=4.9 (95%CI=1.1, 8.9)* |
| Sudden infant death | (Litchfield, Ayres, Jaakkola, & Mohammed, 2018) | Case-crossover | 1 day | OR=1.15 (95%CI=1.02, 1.3)* |

*Significant result based on the 95% confidence interval or the P-value (<0.05)

**The effect of SO2 air pollutant on mortality and hospital admission**

| Outcome | Study | Study design | Lags considered for exposure | RR/IRR/OR/HR/%increase (95%CI or 90%CI or P-value) |
| --- | --- | --- | --- | --- |
| All-cause mortality | (Carey et al., 2013) | Cohort | NA | HR=1.03 (95%CI=1.01,1.05)* |
|  | (Stojic et al., 2016) | Time-series | 0-90 days | %increase=16.13 (95%CI=–2.00, 34.58) |
|  | (Renzi et al., 2017) | Time-series | 0-5 days | %increase= 2.36 (95%CI=1.61, 3.11)* |
|  | (Lyons, Chotirmall, O'Riordan, & Silke, 2014) | Cohort | NA | OR=1.38 (95%CI=1.20, 1.59)* |
| Acute myocardial infarction hospital admission | (Roye et al., 2019) | Time-series | 0 days | RR=1.06 (95%CI=0.94, 1.18) |
| Cardiovascular mortality | (Dehbi et al., 2017) | Cohort | NA | HR=1.05 (95%CI=0.91, 1.22) |
|  | (Carey et al., 2013) | Cohort | NA | HR=1.03 (95%CI=1.01,1.05)* |
|  | (Stojic et al., 2016) | Time-series | 0-90 days | %increase=18.16 (95%CI=3.00, 33.55)* |
| Ischemic stroke hospital admission | (Roye et al., 2019) | Time-series | 0-14 days | RR=0.93 (95%CI=0.85, 1.01) |
| Ischemic stroke mortality | (Roye et al., 2019) | Time-series | 0-14 days | RR=1.09 (95%CI=0.90, 1.31) |
| Lung cancer mortality | (Carey et al., 2013) | Cohort | NA | HR=1.03 (95%CI=0.99,1.06) |
| Pulmonary embolism hospital admission | (de Miguel-Diez et al., 2016) | Case-crossover | NA | OR=1.01 (95%CI=1.00, 1.03) |
| Pulmonary embolism mortality | (de Miguel-Diez et al., 2016) | Case-crossover | NA | OR=1.02 (95%CI=0.97, 1.06) |
| Respiratory mortality | (Carey et al., 2013) | Cohort | NA | HR=1.07 (95%CI=1.04,1.10)* |
|  | (Stojic et al., 2016) | Time-series | 0-90 days | %increase=47.07 (95%CI=–40.17, 142.25) |
| Sudden infant death | (Litchfield, Ayres, Jaakkola, & Mohammed, 2018) | Case-crossover | 1 day | OR=1.01 (95%CI=0.92, 1.12) |

*Significant result based on the 95% confidence interval or the P-value (<0.05)

**The effect of CO air pollutant on mortality and hospital admission**

| Outcome | Study | Study design | Lags considered for exposure | RR/IRR/OR/HR/%increase (95%CI or 90%CI or P-value) |
| --- | --- | --- | --- | --- |
| All-cause mortality | (Atkinson et al., 2016) | Time-series | 1 day | %increase=− 0.79 (95%CI=−1.63, 0.04) |
|  | (Renzi et al., 2017) | Time-series | 0-5 days | %increase=0.12 (95%CI=0.07, 0.17)* |
| Cardiovascular hospital admission | (Samoli et al., 2016) | Time-series | 1 day | %increase=0.95 (95%CI=−0.06, 1.98) |
| Cardiovascular mortality | (Atkinson et al., 2016) | Time-series | 1 day | %increase=− 1.47 (95%CI=−2.94, 0.01) |
| Acute myocardial infarction hospital admission | (Roye et al., 2019) | Time-series | 0 days | RR=1.00 (95%CI=0.87, 1.15) |
| Chest disease hospital admission | (Mekontso et al., 2014) | Time-series | 1-7 days | Coefficient=-0.18 (P-value<0.001)* |
| Pulmonary embolism hospital admission | (de Miguel-Diez et al., 2016) | Case-crossover | NA | OR=1.03 (95%CI=1.02, 1.05)* |
| Pulmonary embolism mortality | (de Miguel-Diez et al., 2016) | Case-crossover | NA | OR=1.01 (95%CI=0.96, 1.06) |
| Respiratory hospital admission | (Samoli et al., 2016) | Time-series | 2 days | %increase=0.21 (95%CI=−0.80, 1.24) |
| Respiratory mortality | (Atkinson et al., 2016) | Time-series | 2 days | %increase=0.41 (95%CI=−1.62, 2.48) |
| Sudden infant death | (Litchfield, Ayres, Jaakkola, & Mohammed, 2018) | Case-crossover | 1 day | OR=1.05 (95%CI=0.95, 1.15) |

*Significant result based on the 95% confidence interval or the P-value (<0.05)

**The effect of cold air temperature on mortality and hospital admission**

| Outcome | Study | Study design | Lags considered for exposure | RR/IRR/OR/HR/%increase (95%CI or 90%CI or P-value) |
| --- | --- | --- | --- | --- |
| All-cause hospital admission | (Hajat et al., 2016) | Time-series | NA | %increase=0.78 (95% CI=0.53, 1.04)* |
| All-cause mortality | (Astrom et al., 2019) | Time-series | 0-21 days | RR=1.28 (95%CI=1.01,1.62)* |
|  | (Astrom et al., 2013) | Time-series | 0-1 days | RR=1.10 (95%CI=1.08,1.13)* |
|  | (Burkart et al., 2013) | Time-series | 0-1 days | %change=0.02 (95%CI=-0.05, 0.09) |
|  | (Burkart et al., 2013) | Time-series | 0-13 days | %change=-0.36 (95%CI=-0.97, 0.26) |
|  | (Hajat et al., 2016) | Time-series | NA | %increase=3.44 (95%CI=3.01, 3.87)* |
|  | (Zeka et al., 2014) | Case-crossover | 1-5 weeks | %increase=6.4 (95%CI=4.8, 7.9)* in Republic of Ireland; %increase=4.5 (95%CI=3.2, 5.9)* in Northern Ireland |
|  | (Zhang et al., 2018) | Time-series | 0 days | RR=0.99 (95%CI=0.985, 0.995)* |
|  | (Breitner et al., 2014) | Time-series | 0-14 days | %change=6.2 (95%CI=1.8,10.8)* |
| Cancer mortality | (Gomez-Acebo et al., 2013) | Case-crossover | 0 days | OR= 4.91 (95%CI=1.85, 13.07)* |
|  | (Gomez-Acebo et al., 2013) | Case-crossover | 6 days | OR=4.02 (95%CI=2.9, 5.58)* |
| Cardiovascular hospital admission | (Ponjoan et al., 2017) | Self-controlled case series | 0 days | IRR=1.20 (95%CI=1.10, 1.30)* |
| Cardiovascular mortality | (Astrom et al., 2019) | Time-series | 0-21 days | RR=1.83 (95%CI=1.31,2.55)* |
|  | (Breitner et al., 2014) | Time-series | 0-14 days | %change=8.9 (95%CI=1.7,16.6)* |
|  | (Breitner et al., 2014) | Time-series | 0-14 days | RR=1.08 (95%CI=1.02, 1.14)* |
|  | (Gomez-Acebo et al., 2013) | Case-crossover | 0 days | OR= 2.72 (95%CI=1.46, 5.08)* |
|  | (Gomez-Acebo et al., 2013) | Case-crossover | 6 days | OR=3.95 (95%CI=3.01, 5.18)* |
|  | (Zeka et al., 2014) | Case-crossover | 1-5 weeks | %increase=5.6 (95%CI=3.5, 7.7)* in Republic of Ireland; %increase=3.9 (95%CI=1.5, 6.3)* in Northern Ireland |
| Cerebrovascular mortality | (Breitner et al., 2014) | Time-series | 0-14 days | RR=1.12 (1.02, 1.22)* |
| Circulatory and respiratory mortality | (Stanisic Stojic et al., 2016) | Time-series | 0-21 days | RR=1.14 (95%CI=0.86, 1.51) |
| Coronary heart disease hospital admission | (Ponjoan et al., 2017) | Self-controlled case series | 0 days | IRR=1.09 (95%CI=0.94, 1.27) |
|  | (Ponjoan et al., 2017) | Self-controlled case series | 3 days | IRR=1.13 (95%CI=1.01, 1.27)* |
| External cause mortality | (Orru & Astrom, 2017) | Time-series | 0-4 days | RR=1.18 (95%CI=1.02, 1.38)* |
| Heart failure hospital admission | (Ponjoan et al., 2017) | Self-controlled case series | 0 days | IRR=1.27 (95%CI=1.12, 1.44)* |
| Ischemic heart disease hospital admission | (Bijelovic et al., 2017) | Time-series | NA | RR=1.51 (95%CI=1.09,2.08)* |
| Ischemic heart disease mortality | (Breitner et al., 2014) | Time-series | 0-14 days | RR=1.04 (95%CI=0.95, 1.14) |
| Myocardial infarction hospital admission | (Bijelovic et al., 2017) | Time-series | NA | RR=1.39 (95%CI=1.01,1.91)* |
| Myocardial infarction mortality | (Gomez-Acebo et al., 2013) | Case-crossover | 0 days | OR= 2.33 (95%CI=0.60, 9.02) |
|  | (Gomez-Acebo et al., 2013) | Case-crossover | 6 days | OR=7.37 (95%CI=3.37, 16.14)* |
| Pulmonary embolism mortality | (de Miguel-Diez et al., 2016) | Case-crossover | NA | OR=1.00 (95%CI=0.99, 1.00) |
| Respiratory mortality | (Astrom et al., 2019) | Time-series | 0-21 days | RR=2.50 (95%CI=0.79,7.86) |
|  | (Breitner et al., 2014) | Time-series | 0-14 days | %change=8.1 (95%CI=−3.0, 20.6) |
|  | (Gomez-Acebo et al., 2013) | Case-crossover | 0 days | OR=3.56 (95%CI=1.43, 8.85)* |
|  | (Gomez-Acebo et al., 2013) | Case-crossover | 6 days | OR=3.44 (95%CI=2.43, 4.86)* |
|  | (Zeka et al., 2014) | Case-crossover | 1-5 weeks | %increase=12.5 (95%CI=8.9, 16.2)* in Republic of Ireland; %increase=11.2 (95%CI=7.1, 15.3)* in Northern Ireland |
| Stroke hospital admission | (Ponjoan et al., 2017) | Self-controlled case series | 0 days | IRR=1.22 (95%CI=1.02, 1.44)* |
|  | (Roye et al., 2019) | Time-series | 0-14 days | RR=1.11 (95%CI=0.93, 1.32) |
| Stroke mortality | (Roye et al., 2019) | Time-series | 0-14 days | RR=1.38 (95%CI=1.13, 1.68)* |
|  | (Zeka et al., 2014) | Case-crossover | 1-5 weeks | %increase=5.0 (95%CI=2.1, 8.0)* in Republic of Ireland; %increase=4.8 (95%CI=0.9, 8.8)* in Northern Ireland |

*Significant result based on the 95% confidence interval or the P-value (<0.05)

**The effect of hot air temperature on mortality and hospital admission**

| Outcome | Study | Study design | Lags considered for exposure | RR/IRR/OR/HR/%increase (95%CI or 90%CI or P-value) |
| --- | --- | --- | --- | --- |
| All-cause hospital admission | (Monteiro et al., 2013) | Time-series | 0-7 days | RR=0.961 (95% CI=0.956,0.967)* |
|  | (Rocklov et al., 2014) | Case-crossover | 0-1 days | ORs= 1.006 (95%CI=0.998, 1.013) |
| All-cause mortality | (Analitis et al., 2014) | Time-series | NA | %increase= 14.6 (95%CI=10.5,18.7)* |
|  | (Aring;strom et al., 2016) | Time-series | 0-2 days | RR=1.18 (95%CI=1.13,1.24)* |
|  | (Aring;strom et al., 2016) | Time-series | 0-10 days | RR=1.13 (95%CI=1.06,1.20)* |
|  | (Astrom et al., 2013) | Time-series | 0-25 days | RR=1.06 (95%CI=1.03,1.08)* |
|  | (Burkart et al., 2013) | Time-series | 0-1 days | %increase=1.30 (95%CI=1.13, 1.66)* |
|  | (Burkart et al., 2013) | Time-series | 0-13 days | %increase=1.39 (95%CI=0.76, 2.01)* |
|  | (Gomez-Acebo et al., 2012) | Time-series | NA | %increase=2.5 (95%CI=1.3, 3.8)* |
|  | (Lopez-Bueno et al., 2019) | Time-series | 0-5 days | RR=1.16 (95%CI=1.03, 1.30)* |
|  | (Miron et al., 2015) | Time-series | 0-7 days | %increase=9.1 (95%CI=8.2, 10.0)* |
|  | (Monteiro et al., 2013) | Time-series | 0-7 days | RR=1.040 (95% CI=1.023,1.056)* |
|  | (Oudin Astrom et al., 2015) | Time-series | NA | RR=1.22 (95%CI=1.18, 1.26)* in Rome; RR=1.08 (95%CI=1.03, 1.12)* in Stockholm |
|  | (Oudin Astrom et al., 2016) | Time-series | 0-2 days | RR=1.18 (95%CI=1.03, 1.34)* |
|  | (Oudin Astrom et al., 2016) | Time-series | 0-10 days | RR=1.12 (95%CI=0.97, 1.29) |
|  | (Rabczenko et al., 2016) | Time-series | 0-7 days | RR=1.093 (95%CI=0.984, 1.214) |
|  | (Ragettli et al., 2017) | Time-series | 0-6 days | RR=1.16 (95%CI=1.09, 1.23)* |
|  | (Rocklov et al., 2014) | Case-crossover | 0-1 days | ORs=1.008 (95%CI=1.001, 1.015)* |
|  | (Willers et al., 2016) | Case-crossover | 2 days | %increase=9.2 (P-value<0.01)* |
|  | (Zafeiratou et al., 2019) | Time-series | 0-3 days | %increase=4.16 (95%CI=3.73, 4.60)* |
|  | (Zhang et al., 2018) | Time-series | 0 days | RR=1.035 (95%CI=1.025, 1.045)* |
|  | (Scortichini et al., 2018) | Time-series | 0-3 days | %increase=19.0 (95%CI=12.0, 22.0)* |
|  | (Breitner et al., 2014) | Time-series | 0-14 days | %increase=8.2 (95%CI=4.6, 12.1)* |
| Cardiovascular hospital admission | (Ponjoan et al., 2017) | Self-controlled case series | 0 days | IRR=0.96 (95%CI=0.85, 1.08) |
|  | (Monteiro et al., 2013) | Time-series | 0-7 days | RR=0.975 (95% CI=0.957, 0.993)* |
| Cardiovascular mortality | (Analitis et al., 2014) | Time-series | NA | %increase=19.9 (95%CI=12.6,27.7)* |
|  | (Breitner et al., 2014) | Time-series | 0-14 days | %increase=8.3 (95%CI=0.7,16.5)* |
|  | (Breitner, Wolf, Peters, & Schneider, 2014) | Time-series | 0-1 days | RR=1.10 (95%CI=1.05, 1.15)* |
|  | (Zafeiratou et al., 2019) | Time-series | 0-3 days | %increase=5.34 (95%CI=4.74, 5.93)* |
|  | (Lopez-Bueno et al., 2019) | Time-series | 0-5 days | RR=1.10 (95%CI=1.04, 1.17)* |
|  | (Miron et al., 2015) | Time-series | 0-7 days | %increase=7.3 (95%CI=6.2, 8.4)* |
| Cerebrovascular mortality | (Breitner et al., 2014) | Time-series | 0-1 days | RR=1.11 (95%CI=1.03, 1.19)* |
| Circulatory and respiratory mortality | (Stanisic Stojic et al., 2016) | Time-series | 0-21 days | RR=1.45 (95%CI=1.20, 1.76)* |
| Congestive heart failure mortality | (Oudin Astrom et al., 2015) | Time-series | NA | RR=1.11 (95%CI=0.99, 1.24) in Rome; RR=1.10 (95%CI=1.02, 1.19)* in Stockholm |
| COPD mortality | (Oudin Astrom et al., 2015) | Time-series | NA | RR=1.25 (95%CI=1.09, 1.43)* in Rome; RR=1.12 (95%CI=0.94, 1.33) in Stockholm |
| Coronary heart disease hospital admission | (Ponjoan et al., 2017) | Self-controlled case series | 0 days | IRR=0.92 (95%CI=0.75, 1.13) |
| Diabetes mortality | (Oudin Astrom et al., 2015) | Time-series | NA | RR=1.25 (95%CI=1.14, 1.37)* in Rome; RR=1.12 (95%CI=1.00, 1.25) in Stockholm |
| External cause mortality | (Orru & Astrom, 2017) | Time-series | 0-1 days | RR=1.24 (95%CI=1.14, 1.35)* |
| Heart failure hospital admission | (Ponjoan et al., 2017) | Self-controlled case series | 0 days | IRR=0.96 (95%CI=0.78, 1.17) |
| Ischemic heart disease hospital admission | (Bijelovic et al., 2017) | Time-series | NA | RR=0.74 (95%CI=0.55,0.99)* |
| Ischemic heart disease mortality | (Breitner, Wolf, Peters, & Schneider, 2014) | Time-series | 0-1 days | RR=1.06 (95%CI=1.01, 1.12)* |
| Mental disorder hospital admission | (Rocklov et al., 2014) | Case-crossover | 0-1 days | ORs=1.011 (95%CI=1.002, 1.019)* |
| Myocardial infarction hospital admission | (Bijelovic et al., 2017) | Time-series | NA | RR=1.07 (95%CI=0.83,1.38) |
| Myocardial infarction mortality | (Oudin Astrom et al., 2015) | Time-series | NA | RR=1.07 (95%CI=0.83, 1.39) in Rome; RR=1.23 (95%CI=1.02, 1.50)* in Stockholm |
| Psychiatric mortality | (Oudin Astrom et al., 2015) | Time-series | NA | RR=1.21 (95%CI=1.06, 1.38)* in Rome; RR=1.33 (95%CI=1.10, 1.61)* in Stockholm |
| Respiratory hospital admission | (Monteiro et al., 2013) | Time-series | 0-7 days | RR=1.018 (95% CI=0.998,1.039) |
| Respiratory mortality | (Analitis et al., 2014) | Time-series | NA | %increase=33.1 (95%CI=20.7, 46.7)* |
|  | (Breitner et al., 2014) | Time-series | 0-14 days | %increase=38.1 (95%CI=20.7, 58.0)* |
|  | (Lopez-Bueno et al., 2019) | Time-series | 0-5 days | RR=1.12 (95%CI=1.03, 1.21)* |
|  | (Miron et al., 2015) | Time-series | 0-7 days | %increase=13.7 (95%CI=11.5, 15.9)* |
|  | (Zafeiratou et al., 2019) | Time-series | 0-3 days | %increase=5.90 (95%CI=4.57, 7.24)* |
| Stroke hospital admission | (Ponjoan et al., 2017) | Self-controlled case series | 0 days | IRR=1.01 (95%CI=0.81, 1.25) |
|  | (Roye et al., 2019) | Time-series | 0-14 days | RR=1.00 (95%CI=0.98, 1.03) |
| Stroke mortality | (Roye et al., 2019) | Time-series | 0-14 days | RR=1.48 (95%CI=1.11, 1.98)* |

*Significant result based on the 95% confidence interval or the P-value (<0.05)

**The effect of air temperature in general on mortality and hospital admission**

| Outcome | Study | Study design | Lags considered for exposure | RR/IRR/OR/HR/%increase (95%CI or 90%CI or P-value) |
| --- | --- | --- | --- | --- |
| All-cause mortality | (Schumann et al., 2013) | Time-series | NA | RR=1.036 (95%CI=0.965, 1.113) |
|  | (Tobias et al., 2014) | Time-series | 0-2 days | %increase=3.3 (95% CI =2.8, 3.7)* |
|  | (Callaly, Mikulich, & Silke, 2013) | Time-series | NA | OR=1.20 (95%CI=1.09, 1.32)* |
|  | (Lyons, Chotirmall, O'Riordan, & Silke, 2014) | Cohort | NA | OR=0.98 (95%CI=0.97, 0.99)* |
| Angina Pectoris hospital admission | (Abrignani et al., 2012) | Time-series | NA | RR=0.992 (95%CI=0.986,0.998)* |
| Chest disease hospital admission | (Mekontso Dessap et al., 2014) | Time-series | 1-7 days | Coefficient=-0.30, (P-value<0.01)* |
| Depression hospital admission | (McWilliams et al., 2014) | Time-series | 0-7 days | Coefficient =0.002, P-value=0.015)* |
| Mania hospital admission | (McWilliams et al., 2014) | Time-series | 0-7 days | Coefficient =0.003, P-value=0.021)* |
|  | (Medici et al., 2016) | Cohort | NA | Coefficient=0.12 (P-value=<0.01)*, |
| Myocardial infarction hospital admission | (Roye et al., 2019) | Time-series | 0 days | RR=1.10 (95%CI=1.03, 1.17)* |
|  | (Wichmann et al., 2013) | Case-crossover | 0-1 days | %increase=-6.1 (95%CI=-11.6, -0.3)* |
|  | (Bhaskaran et al., 2012) | Case-crossover | 1-6 hours | OR=1.019 (95%CI=1.005, 1.033)* |
|  | (Bhaskaran et al., 2012) | Case-crossover | 2-7 days | OR=0.996 (95%CI=0.986, 1.006) |
|  | (Bhaskaran et al., 2012) | Case-crossover | 8-14 days | OR=0.991 (95%CI=0.981, 1.002) |
| Pulmonary embolism hospital admission | (de Miguel-Diez et al., 2016) | Case-crossover | NA | OR=0.98 (95%CI=0.98, 0.99)* |
| Trauma hospital admission | (Roislien et al., 2018) | Time-series | NA | IRR=1.008 (95%CI=1.002, 1.014)* |
| Emergency hospital admission | (Ghirardi, Bisoffi, Mirandola, Ricci, & Baccini, 2015) | Time-series | 0-3 days | %change=3.75 (95%CI=3.01, 4.49)* |

*Significant result based on the 95% confidence interval or the P-value (<0.05)

**The effect of other meteorological exposures on mortality and hospital admission**

| Exposure | Outcome | Study | Study design | Lags considered for exposure | RR/IRR/OR/HR/%increase (95%CI or 90%CI or P-value) |
| --- | --- | --- | --- | --- | --- |
| **Humidity** | Pulmonary embolism mortality | (de Miguel-Diez et al., 2016) | Case-crossover | NA | OR=1.02 (95%CI=0.99, 1.04) |
|  | Pulmonary embolism hospital admission | (de Miguel-Diez et al., 2016) | Case-crossover | NA | OR=1.01 (95%CI=1.00, 1.01) |
|  | Stroke hospital admission | (Santurtun et al., 2017) | Time-series | NA | %decrease=6.5 (P-value = 0.032) |
| **Rainfall** | All-cause mortality | (Schumann et al., 2013) | Time-series | NA | RR=1.004 (95%CI=0.997, 1.010) |
|  | Psychiatric hospital admission | (Medici et al., 2016) | Cohort | NA | Coefficient= 0.02 (P-value=0.58) |
|  |  | (McWilliams et al., 2014) | Time-series | 0-7 days | Coefficient =0.003, P-value=0.001)* |
|  | Sickle cell disease hospital admission | (Piel et al., 2017) | Time-series | 0 days | RR=1.06 (95%CI=1.01, 1.12)* |
| **Sunshine** | Psychiatric hospital admission | (Medici et al., 2016) | Cohort | NA | Coefficient=0.10 (P-value=<0.01)* |
|  |  | (McWilliams et al., 2013) | Time-series | 0-7 days | Coefficient=-0.012 (P-value=0.002)* |
|  |  | (Dominiak et al., 2015) | Time-series | NA | Increasing amounts of sunshine resulted in increased hospitalization (p-value < 0.05) |
|  | Trauma hospital admission | (Roislien et al., 2018) | Time-series | NA | IRR=1.016 (95%CI=1.010, 1.021)* |
| **Snow cover** | Psychiatric hospital admission | (Medici et al., 2016) | Cohort | NA | Coefficient= -0.06 (P-value=0.04)* |
| **Wind speed** | Chest disease hospital admission | (Mekontso Dessap et al., 2014) | Time-series | 1-7 days | Coefficient=0.05 (P-value=0.03)* |
|  | Depression hospital admission | (McWilliams et al., 2014) | Time-series | 0-7 days | Coefficient =-0.003, P-value=0.03)* |
|  | Sickle cell disease hospital admission | (Piel et al., 2017) | Time-series | 0 days | RR=1.06 (95%CI=1.00-1.12) |
| **Wind direction** | Mania hospital admission | (McWilliams et al., 2014) | Time-series | 0-7 days | Coefficient =0.000, P-value=0.023)* |
| **Low air pressure** | Acute myocardial infarction hospital admission | (Bijelovic et al., 2017) | Time-series | NA | RR=0.67 (95%CI=0.47,0.96)* |
| **Low humidity** | Angina Pectoris hospital admission | (Abrignani et al., 2012) | Time-series | NA | RR=1.002 (95%CI=1.001, 1.004)* |
|  | Ischemic heart disease | (Bijelovic et al., 2017) | Time-series | NA | RR=1.38 (95%CI=1.05,1.79)* |
| **High humidity** | Angina Pectoris hospital admission | (Abrignani et al., 2012) | Time-series | NA | RR=0.991 (95%CI=0.985, 0.996)* |
|  | Ischemic heart disease hospital admission | (Bijelovic et al., 2017) | Time-series | NA | RR=0.72 (95%CI=0.52,0.98)* |
| **Maximum pressure** | Mania hospital admission | (McWilliams et al., 2014) | Time-series | 0-7 days | Coefficient =0.002, P-value=0.019)* |
|  | Depression hospital admission | (McWilliams et al., 2014) | Time-series | 0-7 days | Coefficient =0.004, P-value=0.019)* |
|  | Sickle cell disease hospital admission | (Piel et al., 2017) | Time-series | 0 days | RR=0.93 (95%CI=0.88, 0.99)* |
| **Daylight** | Trauma hospital admission | (Roislien et al., 2018) | Time-series | NA | IRR=1.014 (95%CI=1.003, 1.026)* |

*Significant result based on the 95% confidence interval or the P-value (<0.05)
